# Characterization of transgenic mouse models targeting neuromodulatory systems reveals organizational principles of the dorsal raphe

**DOI:** 10.1038/s41467-019-12392-2

**Published:** 2019-10-11

**Authors:** Daniel F. Cardozo Pinto, Hongbin Yang, Iskra Pollak Dorocic, Johannes W. de Jong, Vivian J. Han, James R. Peck, Yichen Zhu, Christine Liu, Kevin T. Beier, Marten P. Smidt, Stephan Lammel

**Affiliations:** 1 Department of Molecular and Cell Biology and Helen Wills Neuroscience Institute, University of California Berkeley, Berkeley, CA 94720 USA; 2 Departments of Physiology and Biophysics, Center for the Neurobiology of Learning and Memory, University of California Irvine, Irvine, CA 92697 USA; 3 Swammerdam Institute for Life Sciences, FNWI University of Amsterdam, Amsterdam, The Netherlands; 4 Present Address: Nancy Pritzker Laboratory, Department of Psychiatry and Behavioral Sciences, Stanford University School of Medicine, Stanford, CA USA

**Keywords:** Neuroscience, Neural circuits

## Abstract

The dorsal raphe (DR) is a heterogeneous nucleus containing dopamine (DA), serotonin (5HT), γ-aminobutyric acid (GABA) and glutamate neurons. Consequently, investigations of DR circuitry require Cre-driver lines that restrict transgene expression to precisely defined cell populations. Here, we present a systematic evaluation of mouse lines targeting neuromodulatory cells in the DR. We find substantial differences in specificity between lines targeting DA neurons, and in penetrance between lines targeting 5HT neurons. Using these tools to map DR circuits, we show that populations of neurochemically distinct DR neurons are arranged in a stereotyped topographical pattern, send divergent projections to amygdala subnuclei, and differ in their presynaptic inputs. Importantly, targeting DR DA neurons using different mouse lines yielded both structural and functional differences in the neural circuits accessed. These results provide a refined model of DR organization and support a comparative, case-by-case evaluation of the suitability of transgenic tools for any experimental application.

## Abstract (web summary)

In addition to serotonin neurons, the dorsal raphe nucleus (DR) also contains dopamine, glutamate, and GABA neurons. Here, the authors systematically compare the neurochemical identity, cell type specificity, anatomical distribution, and connectivity of DR cells targeted by commonly used Cre lines.

## Introduction

The confluence of Cre-driver mouse lines with viral vector technologies has enabled labeling, manipulation, and recording of genetically defined neural circuits. The application of these tools to study neuromodulatory systems has been particularly useful because they provide a handle onto neurons that are interspersed among other cell-types in heterogeneous nuclei^1–6^. A branch of this work has focused on ventral midbrain dopamine (DA) neurons accessed via mouse lines expressing Cre under control of promoters for tyrosine hydroxylase (TH-Cre), an enzyme involved in DA synthesis, or for the DA transporter (DAT-Cre). Indeed, the choice of Cre-driver line proved to be critical for the interpretation of supposedly DA-specific experiments since TH-Cre, but not DAT-Cre, mice were shown to drive transgene expression in cells that lack detectable levels of TH proteins and may not be bona fide DA neurons^7,8^.

Recently, there has been intense interest in the application of Cre-driver lines to study the dorsal raphe nucleus (DR). The DR is best known for its population of serotonin neurons, which can be targeted with mouse lines expressing Cre under the promoter of the serotonin transporter (SERT-Cre) or the enhancer of the transcription factor Pet1 (ePET-Cre)^3,6,9,10^. These neurons send broadly collateralizing axons to most regions of the forebrain^11,12^ and have been shown to play a role in behaviors ranging from locomotion^12–14^ to reward^15–17^, anxiety^18^, allodynia^19^, and social interaction^20,21^. Interestingly, some reports of their functions in these behaviors have been conflicting. For example, some groups have found that activating DR 5HT neurons is reinforcing^16^ and suppresses locomotion^12^, while others have reported the absence of these effects^21–24^, and still others have proposed that these neurons affect locomotion in a state-dependent manner^25^. In addition, DR 5HT neurons show heterogeneous changes in activity in response to motivational stimuli on multiple timescales^22,24,26^, possibly reflecting the functional dynamics of their various inputs^27–29^. A compelling explanation for these inconsistencies could be differential recruitment of functionally distinct subpopulations of DR 5HT neurons, and indeed two projection-defined subsystems of DR 5HT neurons that differ by their expression of the vesicular glutamate transporter VGlut3 (*Slc17a8*) have recently been described^12,30^. Considering that known projection targets of DR 5HT neurons (e.g., striatum) are not represented in this model, further complexity in the DR 5HT system is likely to exist. In addition to 5HT neurons, the DR also contains a population of DA neurons^31^, continuous with the sparse DA neurons of the periaqueductal gray (PAG), which project to the central amygdala (CeA) and bed nucleus of the stria terminalis (BNST)^32–35^. In the last few years, several studies have established roles for DR DA neurons in pain related behaviors^34,36^, social isolation and aversion^35^, arousal and sleep^32^, and associative fear learning^37^. Based on the well-established heterogeneity of VTA DA neurons^38–40^, it is possible that distinct projection patterns of DR DA subpopulations may explain the diverse functionality of these neurons in seemingly disparate behaviors. In the case of DR DA neurons, however, the input/output relationships underlying specific behavioral functions remain largely unknown.

Motivated by the discovery of off-target effects in a mouse line targeting VTA DA neurons^7^, we carried out a comparative analysis of five Cre lines used to study DR 5HT or DA neurons. We find substantial differences in cell-type specificity between DA-targeting lines, and a two-fold difference in penetrance between 5HT-targeting lines. We used these tools to reveal that genetically defined DR populations are anatomically segregated and differ in their afferent and efferent connections. Finally, we show that using different Cre-driver lines to target the same cell population produces structural and functional differences in the neural circuits accessed, as defined by differences in presynaptic inputs and co-transmission of glutamate and dopamine. We thus propose a refined model of DR organization and advocate for a case-by-case evaluation of the suitability of each mouse line for any experimental application.

## Results

### Analysis of Cre lines targeting DR 5HT and DA neurons

**Fig. 1:**
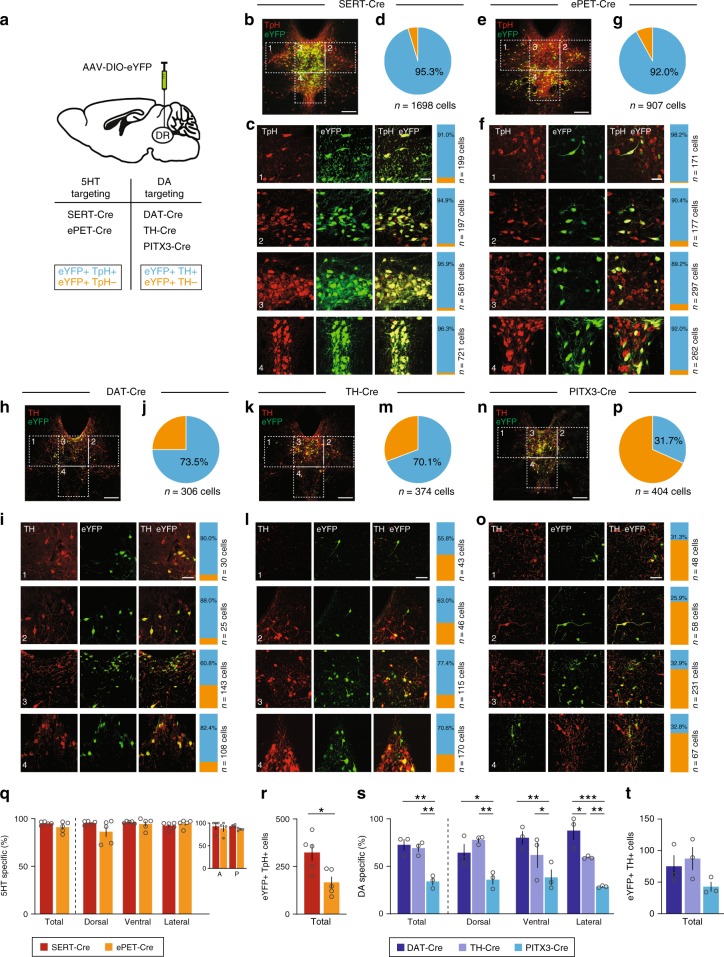
Analysis of transgenic mouse lines targeting DR 5HT and DA neurons. **a** Schematic showing DR injections for different Cre-driver mice. **b** SERT-Cre overview image showing eYFP-positive (eYFP+, green) and tryptophan hydroxylase 2 immunopositive (TpH+, red) neurons in the DR, which is divided into four subregions (scale bar 0.2 mm). **c** Confocal images showing eYFP+ and TpH+ neurons in each subregion. Slice charts indicate percentage of eYFP-positive cells that do (eYFP+ TpH+, blue) or do not co-express TpH (eYFP+ TpH−, orange). Sample images may not correspond to overview (scale bar 50 µm). **d** Pie chart showing percentage of eYFP+ cells that are TpH+ (blue) and TpH− (orange) across all subregions. **e**–**g** Same as **b**–**d**, but using ePET-Cre mice. **h**–**p** Same as **b**–**d**, but using DAT-Cre (**h**–**j**), TH-Cre (**k**–**m**), and PITX3-Cre (**n**–**p**) mice immunostained for tyrosine hydroxylase (TH, red). **q** Bar graph showing average percentage of eYFP+ cells that are TpH+ in 5HT-targeting lines. Dorsal and ventral correspond to subregions 3, 4; Lateral shows pooled data from 1, 2 (total: unpaired *t*-test, t_(4.786)_ = 1.595, *p* = 0.17; subregions: unpaired t-tests followed by Benjamini-Hochberg procedure, t’s < 1.679, p’s > 0.13; *n* = 5 mice per group). Inset shows average percentage of eYFP+ cells that are TpH+ in anterior/posterior DR (two-way ANOVA, F’s < 1.246, p’s > 0.27; *n* = 4 sections each from *n* = 2 mice per group). **r** Bar graph showing average number of eYFP+/TpH+ cells in 5HT-targeting lines (unpaired t-test, t_(7.111)_ = 2.984, *p* = 0.02; *n* = 5 mice per group). **s** Bar graph showing average percentage of eYFP+ cells that are TH-positive (TH+) in DA-targeting lines (total: one-way ANOVA followed by Tukey’s tests, F_(2,6)_ = 20.59, *p* = 0.002; DAT-Cre vs PITX3-Cre, *p* = 0.003; TH-Cre vs PITX3-Cre, *p* = 0.005; subregions: two-way ANOVA on Box-Cox transformed data, main effect of genotype, F_(2,18)_ = 29.14, *p* < 0.0001; dorsal: DAT-Cre vs PITX3-Cre, *p* = 0.01; TH-Cre vs PITX3-Cre, *p* = 0.002. ventral: DAT-Cre vs PITX3-Cre, *p* = 0.003; TH-Cre vs PITX3-Cre, *p* = 0.047. lateral: DAT-Cre vs TH-Cre, *p* = 0.03; DAT-Cre vs PITX3-Cre, *p* < 0.0001; TH-Cre vs PITX3-Cre, *p* = 0.005; no other effects significant, *n* = 3 mice per group). **t** Bar graph showing average number of eYFP+/TH+ cells in DA-targeting lines (one-way ANOVA, F_(2,6)_ = 2.269, *p* = 0.18, *n* = 3 mice per group). Error bars represent SEM; *p < 0.05, **p < 0.01, ***p < 0.001. Data provided as a Source Data file

We first carried out a systematic characterization of the cell-type specificity of five transgenic mouse lines used to target DR 5HT and DA systems (Fig. 1a). With regard to the 5HT system, we examined the SERT-Cre^3^ and ePET-Cre^10^ mouse lines which express Cre under control of the serotonin transporter gene (*Slc6a4*; SERT) or the enhancer of the *Fev* gene encoding Pet1, a transcription factor expressed in 5HT neurons, respectively. For the DA system, we characterized the DAT-Cre^41^, TH-Cre^42^ and PITX3-Cre^43^ mouse lines which express Cre under control of the *dopamine transporter* (*DAT*), *tyrosine hydroxylase* (*TH*), or *PITX3* genes, respectively. *PITX3* codes for a transcription factor involved in the differentiation of midbrain DA neurons, and transgenic lines driven by its promoter have previously been used to study the DA system^31,35,43,44^.

To evaluate cell-type specificity in 5HT neuron targeting lines, we injected a Cre-dependent adeno-associated virus encoding enhanced yellow fluorescent protein (1 μl, AAV-DIO-eYFP) into the DR of SERT-Cre and ePET-Cre mice, then performed immunohistochemistry for tryptophan hydroxylase 2 (TpH, the rate-limiting enzyme in the biosynthesis of 5HT) and divided the DR into four subregions for microscopy and analysis (Fig. 1b, e). Colocalization between eYFP-positive (eYFP+) and TpH−immunopositive (TpH+) neurons was high for both SERT-Cre and ePET-Cre mice (SERT-Cre: eYFP+/TpH+ 95.3%, *n* = 1619/1698 cells from *n* = 5 mice; ePET-Cre: eYFP+/TpH+ 92%, *n* = 834/907 cells from *n* = 5 mice), and there were no significant differences in cell-type specificity between the SERT-Cre and ePET-Cre lines in any DR subregion (Fig. 1c, d, f, g, q). Because DR 5HT neurons are known to be heterogeneous along the rostrocaudal axis, we also compared cell-type specificity in the anterior and posterior DR for a subset of these mice. Again there were no significant differences in the specificity of either line for labeling DR 5HT neurons (SERT-Cre: anterior, eYFP+/TpH+ 92.9 ± 7.1%, *n* = 4 sections from *n* = 2 mice; posterior, eYFP+/TpH+ 93.3 ± 1.7%, *n* = 4 sections from *n* = 2 mice; ePET-Cre: anterior, eYFP+/TpH+ 88.4 ± 7.4%, *n* = 4 sections from *n* = 2 mice; posterior, eYFP+/TpH+ 86.0 ± 1.3%, *n* = 4 sections from *n* = 2 mice) (Fig. 1q, inset). While both Cre lines were similarly specific for DR 5HT neurons, we found that the SERT-Cre-line labeled significantly more TpH+ neurons per mouse, as assessed by the average number of virally-labeled TpH+ cells counted in our analysis, compared to the ePET-Cre line (SERT-Cre: 323.8 ± 43.3 eYFP+/TpH+ cells, *n* = 5 mice; ePET-Cre: 166.8 ± 29.9 eYFP+/TpH+ cells, *n* = 5 mice) (Fig. 1r). Importantly, these differences could not be explained by any limitations of our viral or antibody labeling strategies as AAV-DIO-eYFP injected into the DR of wildtype mice resulted in minimal eYFP expression (Supplementary Fig. 1A–C) and antibody validation experiments showed that TpH staining colocalized with 5HT in nearly every cell analyzed (97.3% of 5HT+ cells were TpH+, 261 out of 268 TpH+ cells from *n* = 1 mouse; and 97.0% of TpH+ cells were 5HT+, 261 cells out of 269 5HT+ cells from *n* = 1 mouse) (Supplementary Fig. 1D–G). To confirm that the difference in labeling observed between the SERT-Cre and ePET-Cre mouse lines was not due to differences in the diffusion of the viral solution, we injected separate cohorts of SERT-Cre and ePET-Cre mice with AAV-DIO-eYFP into the median raphe (MnR), a second serotonergic nucleus (Supplementary Fig. 1H, L). Immunohistochemical analysis revealed slightly lower selectivity for transgene expression in TpH−positive neurons compared to the DR for both lines (SERT-Cre: eYFP+/TpH+ 85.8%, *n* = 641/747 cells from *n* = 3 mice; ePET-Cre: eYFP+/TpH+ 78%, *n* = 160/205 cells from *n* = 3 mice), but again we observed much more efficient viral labeling of TpH+ neurons in SERT-Cre compared to ePET-Cre mice (SERT-Cre: 213.7 ± 58.9 cells; ePET-Cre 53.3 ± 10.7 cells) (Supplementary Fig. 1I–O**)**. Overall, these data indicate that while the cell-type specificities of the SERT-Cre and ePET-Cre mouse lines are comparable, the penetrance of transgene expression between them is different.

Next, we injected AAV-DIO-eYFP (0.3 µl) into the DR of DAT-Cre, TH-Cre, and PITX3-Cre mice and counterstained for TH, the rate-limiting enzyme in the synthesis of DA, to examine their selectivity for targeting DR DA neurons. Colocalization analysis revealed that the majority of eYFP-expressing neurons across the DR were TH-immunopositive (TH+) in DAT-Cre and TH-Cre mice (DAT-Cre: eYFP+/TH+ 73.5%, *n* = 225/306 cells from *n* = 3 mice; Fig. 1h–j; TH-Cre: eYFP+/TH+ 70.1%, *n* = 262/374 cells from *n* = 3 mice; Fig. 1k–m). Strikingly, PITX3-Cre mice showed a much lower measure of TH and eYFP colocalization (eYFP+/TH+ 31.7%, *n* = 128/404 cells from *n* = 3 mice; Fig. 1n–p). Statistical analysis revealed that the PITX3-Cre mouse line was significantly less cell-type-specific for DA neurons compared to the DAT-Cre and TH-Cre lines across all subregions of the DR analyzed (Fig. 1s). While the overall cell-type specificities of the DAT-Cre and TH-Cre mouse lines were similar, the DAT-Cre mouse line was significantly more DA-specific in the lateral wings of the DR suggesting that these two lines may be targeting different, likely partially overlapping, groups of neurons. Comparing the penetrance of these three lines did not reveal any differences (DAT-Cre: 75 ± 17.7 eYFP+/TH+ cells, *n* = 3 mice; TH-Cre: 87.3 ± 18.2 cells, *n* = 3 mice; PITX3-Cre: 42.7 ± 7.7 cells, *n* = 3 mice; Fig. 1t).

In light of the surprisingly poor specificity for TH+ DR cells in PITX3-Cre mice, we repeated our characterization experiments using a larger injection volume (1 µl) to evaluate the full extent of off-target labeling in the DR of this line. We observed more numerous eYFP+ cells in this cohort, but the proportion of labeled neurons that colocalized with TH remained approximately the same (eYFP+/TH+ 40.1%, 381/950 cells from *n* = 3 mice; Supplementary Fig. 1P–R). We also conducted an analogous analysis across seven areas of the ventral midbrain to examine eYFP and TH colocalization in the VTA of PITX3-Cre mice (Supplementary Fig. 1S–Z). We found an anatomical gradient in cell-type specificity such that colocalization was relatively high in the lateral VTA (~80%), but extremely low in midline VTA regions of the caudal and rostral ventral midbrain (including the interfascicular (IF) and rostral linear (RLi) nuclei), which is reminiscent of the expression pattern observed previously in the VTA of TH-Cre mice^7^. Altogether, our results suggest that there are differences in penetrance but not cell-type specificity between these mouse models targeting DR 5HT neurons, while the opposite is true of lines we analyzed targeting DR DA neurons.

### DR cell populations are topographically organized

**Fig. 2:**
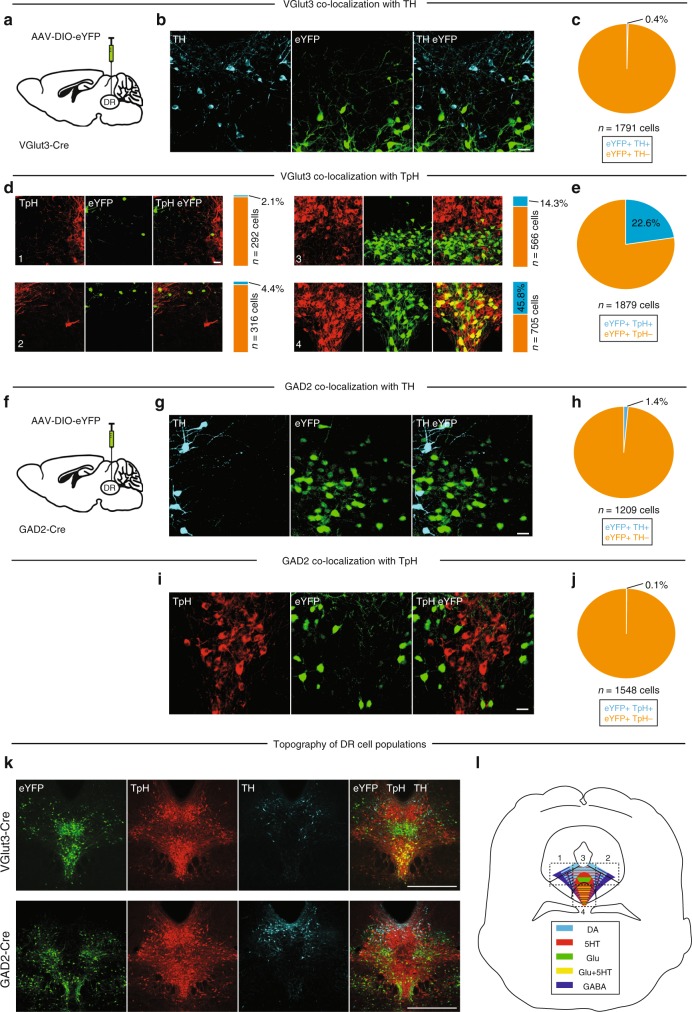
Colocalization analysis between cell-type markers and genetically identified DR neurons. **a** Schematic showing viral injection into the DR of VGlut3-Cre mice. **b** Confocal image showing eYFP-positive (i.e., VGlut3-expressing; eYFP+, green) and TH-immunopositive (TH+, cyan) neurons in the DR (scale bar 50 µm). **c** Pie chart showing total percentage of eYFP+ TH+ (blue) and eYFP+ TH− (orange) cells in the DR. **d** Confocal images showing eYFP+ (i.e., VGlut3-expressing, green) and TpH−immunopositive (TpH+, red) neurons in individual DR subregions (as defined in Fig. 1b). Slice charts indicate percentage of eYFP-positive cells that do (eYFP+ TpH+, blue) or do not co-express TpH (eYFP+ TpH−, orange) in individual DR subregions (scale bar 50 µm). **e** Pie chart showing total percentage of eYFP+ TpH+ (blue) and eYFP+ TpH− (orange) cells when the four DR subregions are considered collectively. **f** Schematic showing viral injection into the DR of GAD2-Cre mice. **g** Confocal image showing TH+ (cyan) and eYFP+ (i.e. GAD2-expressing, green) neurons in the DR (scale bar 50 µm). **h** Pie chart showing total percentage of eYFP+ TH+ (blue) and eYFP+ TH− (orange) cells in the DR. **i** Confocal image showing TpH+ (red) and eYFP+ (i.e., GAD2-expressing, green) neurons in the DR (scale bar 50 µm). **j** Pie chart showing total percentage of eYFP+ TpH+ (blue) and eYFP+ TpH− (orange) cells in the DR. **k** Confocal images showing anatomical distribution of DA (TH+, blue), 5HT (TpH+, red) and eYFP+ (top: VGlut3-expressing, green; bottom: GAD2-expressing, green) neurons in the DR (scale bar 0.5 mm). Note the complementarity in anatomical distribution between VGlut3- and GAD2-expressing DR neurons. **l** Schematic summary depicting anatomical distribution of genetically and/or immunohistochemically defined cell populations in the DR. Data provided as a Source Data file

Motivated by reports of midbrain DA neurons that co-release glutamate^45–47^ or GABA^48^, we next sought to ascertain the degree of overlap between genetically defined cell populations in the DR. We used VGlut3-Cre mice^49^, which express Cre under control of the vesicular glutamate transporter gene *Slc17a8*, and GAD2-Cre mice^50^, which express Cre under control of the *glutamic acid decarboxylase 2* gene involved in GABA biosynthesis, to target DR glutamate and GABA neurons, respectively. We injected AAV-DIO-eYFP (0.3 µl) into the DR of VGlut3-Cre (Fig. 2a) or GAD2-Cre (Fig. 2f) mice and assayed the colocalization of eYFP with TH- and TpH−immunopositive neurons. This analysis revealed that only a very small proportion of VGlut3-expressing neurons contained TH (eYFP+/TH+ 0.4%, *n* = 7/1791 cells from *n* = 3 mice), indicating that the DR consists of distinct, non-overlapping populations of glutamatergic (i.e., VGlut3-expressing, VGlut3+) and dopaminergic cells (Fig. 2b, c). Conversely, there was substantial overlap between VGlut3-expressing cells and TpH, and the anatomical distribution of these double-labeled neurons was strongly biased in favor of the ventral DR. 45.8% of eYFP+ cells co-expressed TpH in the ventral DR (subregion #4, eYFP+/TpH+ 45.8 %, *n* = 323/705 cells) compared to just 14.3% of eYFP+ cells in the dorsal DR (subregion #3, eYFP+/TpH+ 14.3%, *n* = 81/566 cells), and <5% of eYFP+ cells in the lateral DR (subregion #1, eYFP+/TpH+ 2.1%, *n* = 6/292 cells; subregion #2, eYFP+/TpH+ 4.4%, *n* = 14/316 cells). Overall, 22.6% of all eYFP-expressing cells co-expressed TpH (*n* = 424/1879 cells from *n* = 3 mice) and a vast majority of these eYFP+/TpH+ cells were located in the ventral DR (76.2%, *n* = 323/424 cells from *n* = 3 mice) (Fig. 2d, e). These data suggest that VGlut3+ neurons in the DR are subdivided into two anatomically segregated subpopulations: a dorsal group of mostly glutamate-only neurons, and a ventral group where approximately half of glutamatergic cells are also putatively serotonergic. Conversely, immunohistochemical analysis of GAD2-Cre mice suggested that GAD2-expressing neurons are an independent cell population in the DR; only a small proportion of these cells contained detectable levels of TH (eYFP+/TH+ 1.4%, *n* = 17/1209 cells from *n* = 3 mice; Fig. 2 g, h) or TpH (eYFP+/TpH+ 0.1%, *n* = 2/1548 cells from *n* = 3 mice; Fig. 2i, j). Strikingly, comparing injection sites between VGlut3- and GAD2-Cre mice stained for TH and TpH revealed that genetically defined DR cell populations are arranged in a stereotyped, topographical pattern (Fig. 2k, l) that was consistent across the injection sites of all animals studied (Supplementary Fig. 2). Thus, we conclude that the DR is composed of at least five genetically defined, neurochemically distinct and partially anatomically segregated cell types: 5HT (SERT- or ePET-positive) neurons are distributed throughout the DR, GABA (GAD2-positive) neurons are found mostly in the lateral DR, DA (DAT-positive) neurons are found mostly in proximity to the aqueduct, and glutamatergic (VGlut3-positive) neurons are concentrated in the medial DR and subdivided into dorsal and ventral groups with minimal and significant co-expression of 5HT cell markers, respectively (Fig. 2k, l, Supplementary Fig. 2). Strikingly, analysis of the axon projection targets from genetically defined DR populations showed that all groups send convergent input to the VTA, but they preferentially project to distinct subnuclei of the amygdala (Supplementary Fig. 3A). In particular, DR 5HT neurons project most densely to the BLA, while DR DA neurons project preferentially to the lateral CeA, and DR glutamate and GABA neurons mainly innervate the capsular and medial parts of the CeA, respectively (Supplementary Fig. 3B).

### Tracing DR circuits accessed with different Cre-driver lines

**Fig. 3:**
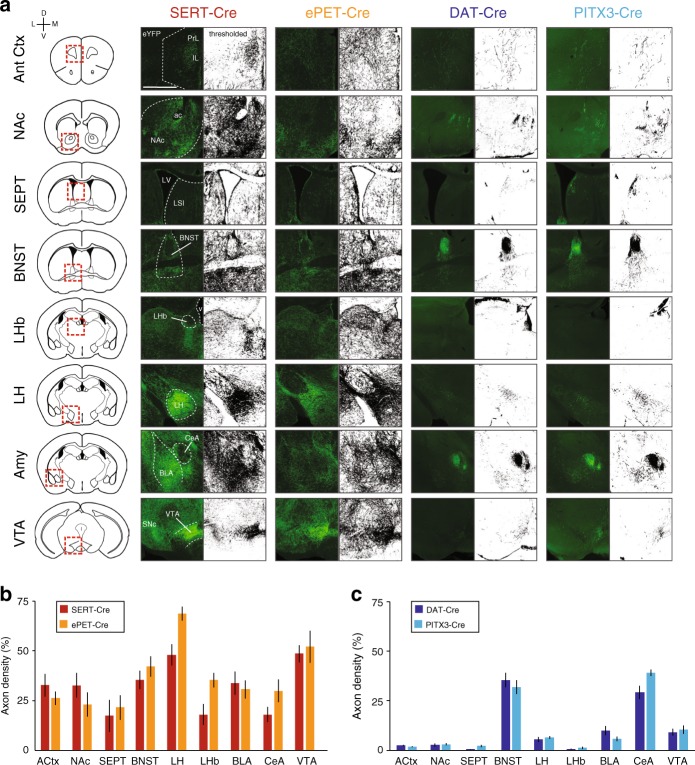
Projections of DR neurons in different transgenic mouse lines. **a** Left: Schematic of each anatomical region analyzed. Right: Fluorescence images showing eYFP-labeled terminals in different brain regions from DR neurons targeted using SERT-Cre, ePET-Cre, DAT-Cre, and PITX3-Cre mouse lines adjacent to thresholded and binarized versions of the same images (scale bar 0.5 mm). **b** Bar graph showing the percentage of each target region that is covered by terminals from DR neurons labeled in SERT-Cre and ePET-Cre mouse lines (two-way ANOVA, main effect of region, F(8,90) = 10.97, *p* < 0.0001; no main effect of genotype or region by genotype interaction, F’s < 3.788, p’s > 0.055; *n* = 6 sections for each region from *n* = 2 mice in each group). **c** Same as in **b** but experiments were performed in DAT-Cre and PITX3-Cre mice (two-way ANOVA, region by genotype interaction F(8,90) = 2.085, *p* = 0.0453; *n* = 6 sections for each region from *n* = 2 mice in each group). Error bars represent SEM. Data provided as a Source Data file

Next, we examined the efferent and afferent connectivity of DR 5HT and DA neurons. Based on the results of our Cre-line characterization experiments, we hypothesized that the anatomical organization of these neural circuits could depend on both the neurochemical identity of the target population and the mouse line used to target it. To test this, we examined axon density in eight brain regions known to be strongly innervated by the DR^51^: VTA, lateral hypothalamus (LH), lateral habenula (LHb), amygdala (Amy), bed nucleus of the stria terminalis (BNST), septum (SEPT), nucleus accumbens (NAc), and anterior cortex (Ant Ctx). Specifically, we injected AAV-DIO-eYFP into the DR of SERT-Cre and ePET-Cre mice (1 μl to label neurons throughout the entire extent of the DR, *n* = 2 mice per group) and of DAT-Cre and PITX3-Cre mice (0.3 µl to minimize virus leak into the VTA, *n* = 2 mice per group), immunostained for GFP, and imaged sections on a slide scanning microscope. Images were background subtracted, thresholded, and binarized to create black-and-white images showing the organization of axon fibers from DR cell populations in each target region (Fig. 3a), and axon density was quantified as the percentage of black pixels in each region of interest. Analysis of variance showed that there are no significant differences in axon density between the SERT-Cre and ePET-Cre lines (Fig. 3b), but there is a significant genotype-by-target region interaction between the axon densities of the DAT-Cre and PITX3-Cre mouse lines (Fig. 3c). While post-hoc tests comparing the means between these two groups at individual target regions were not significant after correcting for multiple comparisons, this suggests that the global patterns of axon projections may be subtly different between groups of DR DA neurons targeted using different transgenic mouse lines. Consistent with previous reports^12,34,35,37^ we found that DR 5HT neurons provide relatively dense innervation to all targets studied, while DR DA neurons send fairly specific projections to the CeA and BNST, and especially to the lateral and oval sub-compartments of those nuclei, respectively.

**Fig. 4:**
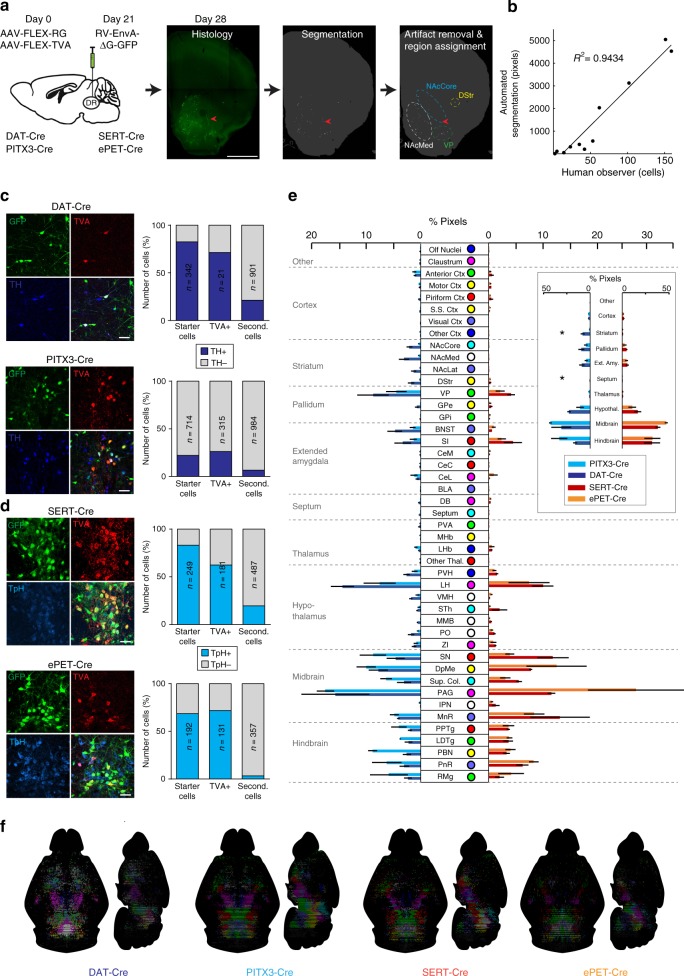
Monosynaptic inputs onto DR neurons in different transgenic mouse lines. **a** Left: Schematic of viral injections for tracing monosynaptic inputs onto DR neurons using DAT-Cre, PITX3-Cre, SERT-Cre or ePET-Cre mice. Right: Fluorescence image showing GFP-positive (GFP+) cells in the striatum before (left) and after automated segmentation by pixel intensity (middle, positive pixels in white). Segmented pixels were semi-automatically assigned to anatomical structures (right); e.g. dorsal striatum (DStr), ventral pallidum (VP), nucleus accumbens medial shell (NAcMed). Red arrow indicates a manually removed image artifact (right, scale bar: 1 mm). **b** Graph showing correlation between manually counted input neurons and automated segmentation procedure. **c** Confocal images of DR starter cell populations in DAT-Cre (top) and PITX3-Cre (bottom) mice (green: RV-∆G-GFP, red: TVA-mCherry, blue: TH; scale bars 50 µm). Bar graphs (right) show colocalization between TH and starter cells (GFP+ TVA-mCherry-positive [mCherry+] cells), TVA+ non-starter cells (GFP-negative [GFP-] mCherry+ cells), and secondary cells (GFP+ mCherry- cells). **d** Confocal images of DR starter cells in SERT-Cre (top) and ePET-Cre (bottom) mice (green: RV-∆G-GFP, red: TVA-mCherry, blue: TpH; scale bars 50 µm). Bar graphs (right) show colocalization between TpH and starter cells, TVA+ non-starter cells, and secondary cells. **e** Bar graph showing quantification of inputs onto DR neurons targeted using DAT-Cre (*n* = 4; dark blue), PITX3-Cre (*n* = 3; light blue), SERT-Cre (*n* = 3; red) and ePET-Cre (*n* = 3; orange) mice. Data are presented as a percentage of total inputs (% pixels) counted in each brain. Inset shows data pooled into anatomical subdivisions indicated by dashed gray lines (two-way ANOVA on Box-Cox transformed data for DA-targeting lines: genotype-by-region interaction, F(9,50) = 3.336, *p* = 0.003; striatum: DAT-Cre vs PITX3-Cre, *p* = 0.002; septum: DAT-Cre vs PITX3-Cre, *p* = 0.04; two-way ANOVA on Box-Cox transformed data for 5HT-targeting lines: main effect of region, F(9,40) = 68.71, *p* < 0.0001; no main effect of genotype, no genotype-by region interaction, F’s < 2.351, p’s > 0.13, no other tests significant). Error bars represent SEM, *p < 0.05. See methods for definitions of anatomical abbreviations. **f** Horizontal and sagittal of brain-wide inputs to DR neurons in a representative mouse from each transgenic mouse line. Colors correspond to structures shown in **e**. Data provided as a Source Data file

To examine differences in inputs between DR DA or 5HT neurons targeted using different mouse lines, we combined monosynaptic rabies tracing with semi-automated brain mapping software^52^. Sections were imaged on a slide scanning microscope and GFP-positive cells were identified based on a pixel-intensity threshold. Image artifacts were manually removed, segmented pixels were assigned to anatomical regions based on the mouse brain atlas^53^ (Fig. 4a), and inputs are reported as the percentage of pixels per area out of the total pixels counted in each brain. Because this analysis is based on an intensity threshold, it may slightly overestimate the number of neurons in regions where fluorescent neurites are dense, but importantly a validation experiment showed a strong correlation between manually counted cells and pixels counted by our software (R^2^ = 0.9434; Fig. 4b).

We injected DAT-Cre, PITX3-Cre, SERT-Cre and ePET-Cre mice in the DR with AAVs encoding the cellular receptor for subgroup A avian leukosis viruses and the rabies virus glycoprotein (AAV-FLEX-TVA-mCherry and AAV-FLEX-RG, respectively; 0.8 µl, 1:1), followed 3 weeks later by injection of EnvA-pseudotyped, glycoprotein-deficient rabies virus expressing GFP (RV-EnvA-ΔG-GFP; *n* = 3–4 mice per line; Fig. 4a). Analysis of starter cells (i.e., GFP- and TVA-positive cells) in the DR of DAT-Cre mice showed that the majority of starter cells were TH-immunopositive (82.5% TH+), whereas in PITX3-Cre mice, most starter cells were TH-immunonegative (77.9% TH−; Fig. 4c). In contrast, the proportion of starter cells that were TpH−positive was similar in SERT-Cre and ePET-Cre mice (83.1% and 70.3%, respectively; Fig. 4d). Likely due to the documented sensitivity of pseudotyped RV for labeling neurons that express trace, background levels of the TVA receptor, the degree of cell-type specificity observed here was slightly lower compared to the results of our previous Cre-line characterization experiments^54^. However, the trends in specificity (ePET-Cre ≈ SERT-Cre; DAT-Cre > PITX3-Cre) were consistent with our previous data, and control experiments in wildtype mice (C57Bl/6; *n* = 3 mice) and in SERT-Cre mice injected with RV-EnvA-∆G-GFP but not FLEX-TVA or FLEX-RG (*n* = 3 mice) yielded very few (<10 cells per animal) GFP-positive cells in the DR (Supplementary Fig. 4A–D) thus confirming the validity of our genetic targeting approach. Additional control experiments where only AAV-FLEX-TVA-mCherry and RV-EnvA-∆G-GFP were injected confirmed that no transsynaptic spread is detectable in the absence of RG (Supplementary Fig. 4E–I).

Overall, our experiments using rabies virus revealed that DR 5HT neurons targeted by the SERT- and ePET-Cre lines receive similar presynaptic inputs (Fig. 4e). By contrast, DR DA neurons targeted using DAT-Cre mice receive a greater proportion of their input from the striatum and septum compared to DR DA neurons targeted by PITX3-Cre mice (Fig. 4e, f).

**Fig. 5:**
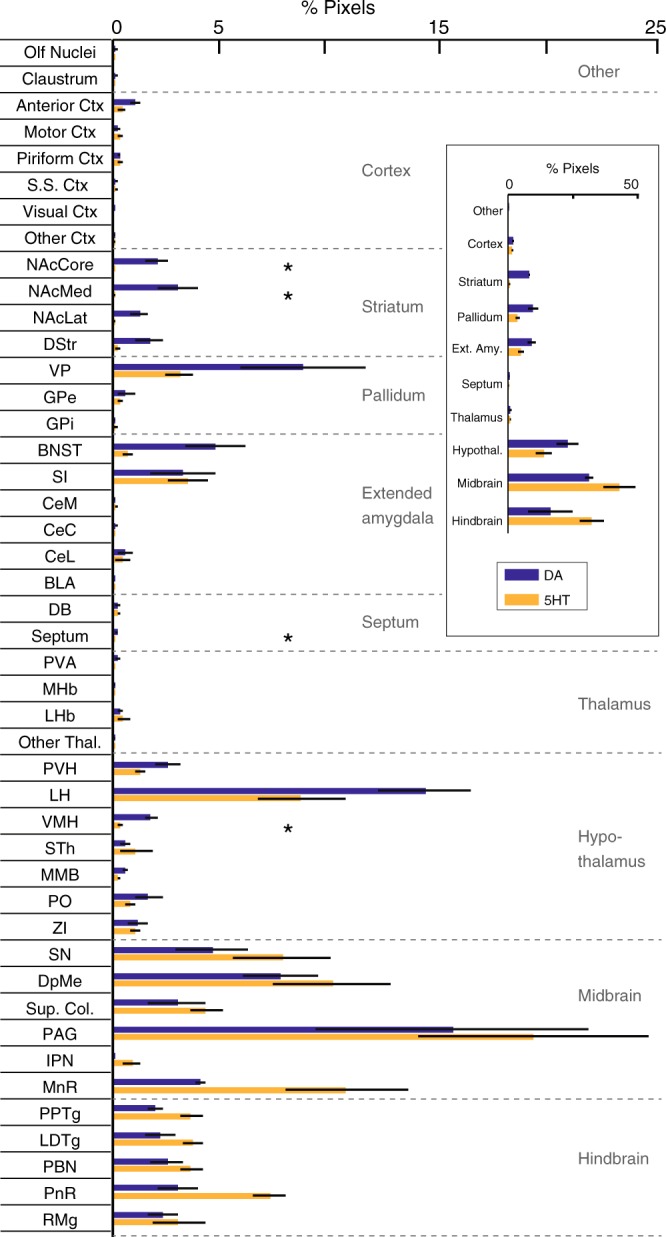
Differences in monosynaptic inputs onto DA and 5HT DR neurons. Bar graph showing quantification of inputs onto DA (*n* = 4 mice, dark blue) and 5HT (*n* = 6 mice, yellow) DR neurons. DA group data is from DAT-Cre mice only to avoid the off-target effects of the PITX3-Cre line. Data from SERT-Cre and ePET-Cre lines were pooled for the 5HT group due to similar specificity for 5HT neurons. Data are presented as a percentage of total input (% pixels) counted in each individual brain (means were compared directly because data did not meet the equal variance assumption for two-way ANOVA [see statistical methods for details]; unpaired t-tests followed by Benjamini-Hochberg procedure, nucleus accumbens core [NAcCore]: t_(8)_ = 4.832, *p* = 0.029; nucleus accumbens medial shell [NAcMed]: t_(8)_ = 4.125, *p* = 0.037; septum: t_(8)_ = 4.400, *p* = 0.034; ventromedial hypothalamus [VMH]: t_(8)_ = 5.679, *p* = 0.021). Inset shows a summary of input data pooled into major anatomical subdivisions indicated by dashed gray lines and labels. Error bars represent SEM, *p < 0.05. See methods for definitions of anatomical abbreviations. Data provided as a Source Data file

Given that monosynaptic inputs onto DR DA neurons have not previously been reported, we then compared pooled data from the similarly cell-type-specific ePET- and SERT-Cre lines to the input data for the DAT-Cre group. In agreement with previous work, we found that major inputs onto DR 5HT neurons originate from the PAG, deep mesencephalic nucleus (also known as the midbrain reticular nucleus), pontine reticular nucleus, and lateral hypothalamus^27–29^. While DR DA and 5HT neurons receive input from qualitatively similar brain regions, DR DA neurons receive quantitatively more input from subregions of the nucleus accumbens (NAc), septum, and ventromedial hypothalamus (VMH) compared to DR 5HT neurons (Fig. 5). Thus, both the neurochemical identity of starter cells and the specific Cre-driver line used to access them are critical components that define the circuit architecture of DR neurons targeted using viral strategies.

### Optogenetic dissection of the DR/PAG → CeA pathway

Recent studies suggested that DR/PAG DA neurons projecting to CeA may co-release glutamate and DA^35,37^. Specifically, using TH-Cre mice, Groessl et al^37^. demonstrated that optogenetic activation of the DR/PAG → CeA pathway induced excitatory postsynaptic currents (EPSCs) in CeA neurons, presumably via synaptically released glutamate. We decided to re-evaluate these findings in light of the regional differences in cell-type specificity that we observed between TH-Cre and DAT-Cre mice in the VTA^7^ and DR (Fig. 1k–m). Because VGlut3 is selectively expressed in the DR^55^, but DR DA neurons do not express VGlut3 (Fig. 2b, c), we hypothesized that DA and glutamate co-release in the CeA must originate from VGlut2-expressing DA neurons in the nearby PAG. Alternatively, the use of TH-Cre mice, in which Cre expression extends beyond the targeted DA cell population (especially in the lateral DR), may lead to the optogenetic manipulation of unintentionally targeted glutamate cells in the DR and/or PAG, which could have been erroneously interpreted as DA and glutamate co-release.

**Fig. 6:**
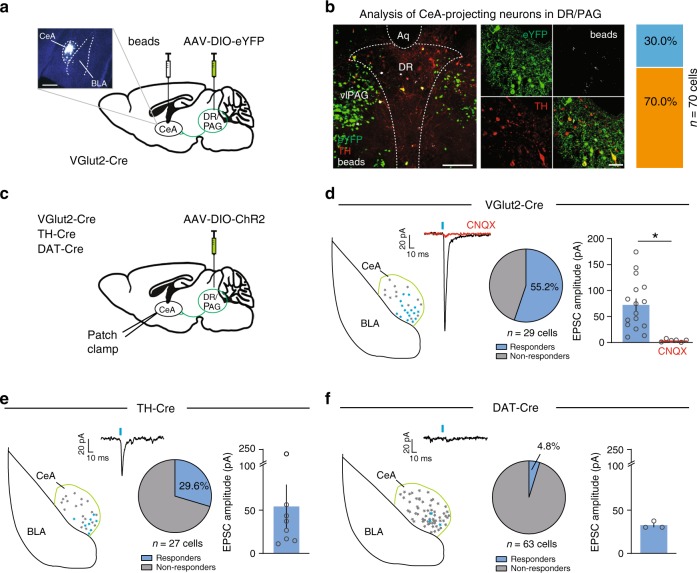
Optogenetic dissection of the DR/PAG → CeA pathway. **a** Schematic showing retrograde labeling of central amygdala (CeA) projecting neurons in the dorsal raphe/periaquecductal gray (DR/PAG) using VGlut2-Cre mice. Inset shows sample injection site of retrobeads (white) in the CeA (scale bar 0.5 mm). **b** Analysis of colocalization between eYFP-positive (i.e., eYFP+, VGlut2 expressing; green) and TH-immunopositive (i.e., TH+, putatively dopaminergic; red) CeA-projecting (i.e., beads+, white) cell populations in the DR/PAG. Left: Confocal image showing overview of DR/PAG in VGlut2-Cre mice (scale bars 0.5 mm). Middle: Higher magnification image. Right: Slice charts showing percentage of beads+ and eYFP+ cells that co-express TH (eYFP+/beads+/TH+, blue) or lack expression of TH (beads+/eYFP+/TH−, orange) in the DR/PAG (scale bars 50 µm). **c** Schematic of experimental design to analyze functional connectivity of DR/PAG inputs to CeA neurons using DAT-Cre, TH-Cre and VGlut2-Cre mice. **d** Sample traces from whole-cell recordings at −70 mV showing EPSCs generated by light stimulation of DR/PAG_VGLUT2_ inputs to CeA neurons (red trace: sample of mean EPSC after CNQX application; scale bars: 20 pA/10 ms). Schematics shows localization of the recorded neurons in the CeA that responded (blue) and did not respond (gray) to light stimulation. Pie chart shows percentage of responders (blue) and non-responders (gray). Bar graph showing mean EPSC amplitudes for the cells that responded to light stimulation before (blue) and after (red) bath application of 10 μM CNQX (paired *t*-test, t_(5)_ = 3.835 *p* = 0.0122, *n* = 6 cells; *p < 0.05; error bars represent SEM). **e**, **f** Same as in **d**, but experiments were performed in TH-Cre (**e**) and DAT-Cre (**f**) mice. Data provided as a Source Data file

To examine the cellular identity of CeA-projecting neurons in the DR and PAG, we injected VGlut2-Cre mice with fluorescent retrobeads (100 nl) into the CeA and AAV-DIO-eYFP (0.3 µl) into the DR/PAG (Fig. 6a). We found that 21% of retrogradely labeled neurons in the DR/PAG were TH-immunopositive (*n* = 25/119 cells from *n* = 2 mice), confirming the presence of a dopaminergic projection in addition to projections from non-DA neurons. Of the 70 retrogradely labeled cells that expressed eYFP (i.e. VGlut2-expressing neurons) though, only 30% were TH-immunopositive (Fig. 6b) indicating that only a small population of neurons are equipped to putatively co-release DA and glutamate. Considering the non-DA-specific labeling pattern we observed in the lateral DR of TH-Cre mice, we sought to evaluate whether functional glutamate release in the DR/PAG → CeA pathway originates from the few DA neurons equipped for glutamate co-release or from TH-negative glutamate neurons unintentionally recruited by the TH-Cre mouse line. To test this, we injected a Cre-dependent AAV expressing channelrhodopsin-2 (AAV-DIO-ChR2; 0.3 µl) into the DR/PAG of VGlut2-Cre (*n* = 3), TH-Cre (*n* = 2) or DAT-Cre (*n* = 4) mice (Fig. 6c). Whole-cell recordings from CeA neurons revealed that light pulses selectively stimulating VGlut2 ChR2 fibers in the CeA evoked robust EPSCs in ~55% of recorded neurons (72.4 ± 12.2 pA, *n* = 16/29 cells) and these were blocked by an AMPA (α-amino-3-hydroxy-5-methyl-4-isoxazole propionic acid) receptor antagonist (CNQX: 3.8 ± 1.1 pA, *n* = 6 cells), thus revealing a direct excitatory input to the CeA (Fig. 6d). While light stimulation of TH-Cre ChR2 fibers in the CeA evoked EPSCs in ~30% of the recorded CeA neurons (54.2 ± 25.4 pA, *n* = 8/27 cells; Fig. 6e), light pulses almost never evoked detectable EPSCs in CeA neurons recorded from DAT-Cre mice. Fewer than 5% of cells recorded in DAT-Cre mice responded to optical stimulation (25.9 ± 8.2 pA, *n* = 3/63 cells; Fig. 6f), indicating that DR/PAG DA neurons targeted by DAT-Cre mice almost completely lack the ability to generate light-evoked excitatory responses in CeA neurons. These results illustrate how the choice of transgenic mouse line used to access a targeted population of neurons can influence the interpretation of studies investigating their functional properties.

## Discussion

The goals of this study were: (1) to evaluate Cre-driver lines used to target neuromodulatory populations of the DR; and (2) to leverage these tools to dissect DR circuitry with an emphasis on how the choice of mouse model could lead to differences in the neural circuitry studied.

Consistent with a previous study^23^, we found that SERT-Cre and ePET-Cre mice exhibit similar cell-type specificity, but our data suggest that the SERT-Cre-line labels ~2–3 times more 5HT neurons. This trend was consistent across the DR and MnR, which is in line with work showing that a subset of 5HT neurons (~30%, termed *Pet1*-resistant) develop independently of *Pet1* expression^56,57^. The slightly lower cell-type specificity observed in the MnR of ePET-Cre mice is in agreement with a report of another transgenic mouse line based on the *Pet1* gene showing reduced specificity for 5HT neurons in serotonin cell groups B5 and B8^58,59^, corresponding to the MnR^53^. The difference in penetrance between these two lines raises the possibility that ePET-Cre mice could be labeling a specific subset of DR 5HT neurons. While further work will be necessary to answer this conclusively, our experiments showed that the ePET-Cre^-^ line labeled 5HT neurons without an obvious bias for any DR subregion and the connectivity of labeled neurons between the ePET-Cre and SERT-Cre mouse lines was largely similar. Previous studies argued that *Pet1-*resistant 5HT neurons preferentially innervate the BLA and VMH, which predicts that these regions would be more densely innervated by the SERT-Cre-line compared to the ePET-Cre-line^58,59^. While our analysis of axon projections did not include the VMH, we observed a small trend in the predicted direction when we compared BLA innervation between SERT-Cre and ePET-Cre mice, but this did not reach statistical significance. Thus, our data mostly argue against the idea that the ePET-Cre-line is labeling an anatomically specific subset of 5HT neurons compared to the SERT-Cre line, though it is possible that the SERT-Cre-line may also label a small population of neurons not targeted in ePET-Cre mice. Overall these differences have important implications for the interpretation and design of cell-type-specific manipulations of 5HT neurons since they suggest that experiments performed in SERT-Cre and ePET-Cre mice would likely recruit different proportions of the DR or MnR 5HT systems, potentially leading to divergent behavioral or physiological responses. The surprising finding that SERT-Cre and ePET-Cre mice did not show any significant differences in our comparison of their axonal projections despite their different penetrance could be due to the extremely broad collateralization patterns of axons from DR 5HT neurons^12^. Because our experiment included immunostaining for GFP to amplify the signal from axon branches, there may be a ceiling effect in regions that receive dense projections from DR 5HT neurons, which could have made it technically difficult to resolve differences between the SERT-Cre and ePET-Cre lines’ projection densities. This limitation would be less likely to affect our analysis of DR DA neuron efferents given that their axons do not collateralize nearly as broadly.

On the other hand, we found substantial differences in specificity between DAT-, TH-, and PITX3-Cre mice targeting DR DA neurons. All three lines showed notable levels of Cre expression in TH-immunonegative DR neurons, but our data indicate that PITX3-Cre mice are less cell-type-specific than both DAT-Cre and TH-Cre mice, while DAT-Cre mice offer the best cell-type specificity in the lateral DR. These differences could theoretically be explained by some Cre+ neurons expressing TH at levels below the immunodetection limit, or by limitations associated with our immunostaining or imaging procedures. However, this is unlikely to account for our observations for several reasons. First, the qualitative observation of Cre+/TH− neurons in PITX3- and TH-Cre mice has been made by others using different reagents and imaging strategies^43,44,60^. Second, the distribution of eYFP+/TH− cells near the DR in TH-Cre and PITX3-Cre mice extended into the lateral PAG and DpME regions where very few and no DA neurons are known to reside, respectively, and this was not observed in the more cell-type-specific DAT-Cre-line. Third, the distribution of Cre+ cells in the midbrain of TH-Cre and PITX3-Cre mice extended into regions not known to contain DA neurons (e.g., interpeduncular nucleus)^7^. Together, these lines of evidence support the interpretation that the TH-Cre and PITX3-Cre mouse lines exhibit Cre expression in TH-immunonegative VTA neurons, and that the same is true in the DR for all three mouse lines albeit to a lesser extent in DAT-Cre mice. The neural circuits accessed via different Cre lines targeting DR DA neurons also differed with respect to their inputs. While we cannot make strong conclusions about whether these differences in connectivity could be due to the differences in the cell-type specificities of these mouse lines, heterogeneity in the starter cells they label, or both, these results indicate that the choice of driver line used to access neuromodulatory populations can lead to systematic anatomical differences in the circuits accessed.

Notably, the pattern of TH-immunonegative cell labeling in PITX3-Cre mice was similar to that observed in the midbrain of TH-Cre mice;^7^ prominent Cre expression was observed in the IF and RLi nuclei. The finding that these cells are labeled by mouse lines under the control of genes expressed early (TH, PITX3), but not later in the development of the mesencephalon (DAT), suggests that some eYFP+/TH− neurons may have transiently expressed DA-related genes in development but subsequently lost the ability to synthesize DA^43^. Thus, in adult TH-Cre and PITX3-Cre mice, Cre may be present in cells that do not contain TH mRNA, as has been observed in the RLi nucleus of PITX3-Cre mice^44^, or in cells that contain TH mRNA but not TH protein, as has been found in the IPN of TH-Cre mice^7^. Importantly, while Cre-expressing RLi nucleus and IPN neurons lack the molecular machinery to produce DA, many RLi nucleus neurons express VGlut2 suggesting that these cells could be functionally glutamatergic^44^. Thus, it is essential to detect these off-target Cre-expression events because this may confound the interpretation of supposedly cell-type-specific manipulations, which may be recruiting non-DA neurons^61^. In this way, our results highlight some caveats associated with using Cre-driver mouse lines to target neuromodulatory cell populations; including transgene expression in cells that transiently expressed the gene of interest during development, in populations where the gene of interest is transcribed but not translated, and in neurons that translate the protein of interest in the absence of other machinery required for transmission of the neuromodulator in question.

Moreover, a recent study using TH-Cre mice suggested that DR/PAG DA neurons co-release DA and glutamate^37^. While there is evidence that DR/PAG terminals release DA in the CeA in vivo^35^, our results question whether the same population is also releasing glutamate. We found that the majority (~70%) of DR/PAG neurons projecting to the CeA are glutamatergic (i.e., VGlut2-positive) and lack detectable levels of TH protein, so TH-immunopositive neurons labeled by VGlut2 reporter lines (which have been described previously in the DR/PAG^34,35^) represent only a small subset (~30%) of CeA-projecting DR/PAG neurons. Importantly, stimulation of DR/PAG DA neurons produced reliable EPSCs in CeA cells of VGlut2-Cre and TH-Cre, but not DAT-Cre, mice. One possible explanation for the observation of TH+ neurons labeled by VGlut2 reporter mice may be that these cells could express VGlut2 mRNA without synthesizing functional VGlut2 protein. In light of the prominent non-DA-specific expression patterns observed in the TH-Cre mouse line, however, it is conceivable that the EPSCs recorded in CeA neurons of these mice are attributed to unintentional expression of ChR2 in DR/PAG glutamate neurons. Nevertheless, the possibility of DA and glutamate co-release in the CeA cannot be excluded entirely as another study detected light-evoked EPSCs in CeA neurons following injection of a ChR2-enconding virus into the DR of DAT-Cre mice^35^. However, given that the proportion of neurons that co-express VGlut2 and TH is greater in the adjacent caudal linear (CLi) nucleus^62^ compared to the DR, this finding could also be explained by virus leak into the CLi nucleus from DR-targeted injections. Thus, while there is evidence that some DA neurons in the ventral midbrain co-release glutamate^45–47^ or GABA^48^, additional studies examining this phenomenon in the DR/PAG are necessary.

Collectively, our data highlight the importance of individually assessing the strengths and weaknesses of each transgenic mouse line. While our data indicate that the DAT-Cre and SERT-Cre lines may be the best available tools for targeting DR DA and 5HT neurons in adult mice, the ePET-Cre, TH-Cre, and PITX3-Cre lines may be well suited for other applications. For example, the TH-Cre-line labels mesocortical VTA DA neurons that express low levels of DAT and are therefore likely to be under sampled by DAT-Cre mice^63,64^. Similarly, the ePET-Cre-line may be a preferable tool for studies of DR 5HT neurons that require sparse labeling. Furthermore, the PITX3-Cre and ePET-Cre lines label DA or 5HT neurons, respectively, earlier during development compared to DAT-Cre and SERT-Cre mice^44,65^ likely making them powerful tools for studying the assembly of these circuits. Thus, we interpret our results as evidence in favor of a diligent case-by-case evaluation of the suitability of each transgenic mouse line for accomplishing a specific scientific goal.

Finally, we used these mouse lines to show that genetically defined DR 5HT, DA, GABA, and glutamate populations are arranged in a topographical pattern. This result is in agreement with reports describing complementary distribution patterns of DR glutamate and GABA neurons in the rat^23,66^, the lateral distribution of DR GABA cells in mice^23,25,29,67^, the ventral distribution of VGlut3+/TpH+ DR neurons in rats^66^, and the co-expression of VGlut3 in cortically-projecting 5HT neurons of the mouse ventral DR^12^. To the best of our knowledge though, our description of mouse DR organization is the first detailed analysis of the distribution and overlap for all four major cell types. We add to this model a comparative analysis of projections from DR DA and 5HT neurons, and the first description of inputs onto DR DA neurons. Comparing this result with input tracing data for VTA DA neurons^52^ suggests that while both groups sample from qualitatively similar inputs, DR DA neurons likely sample a greater proportion of inputs from the BNST. Given that the BNST is a major projection target of DR DA neurons, this suggests that there is strong reciprocal connectivity in this circuit, which may be important for understanding the functional roles of these neurons. Thus, our results provide the anatomical basis for future investigations of DR circuitry and illustrate how the systematic evaluation and application of transgenic mouse lines advances our understanding of anatomical and functional diversity in heterogeneous brain regions.

## Methods

### Subjects

The following mouse lines (8–16 weeks old) were used for experiments: C57BL/6 J mice (Jackson Laboratory, stock number: *000664*), DAT::IRES-Cre (Jackson Laboratory, stock number: *006660*, strain code: B6.SJL-Slc6a3tm1.1(cre)Bkmn/J), VGLUT2::IRES-Cre (Jackson Laboratory, stock number: *016963*, strain code: Slc17a6tm2(cre)Lowl/J), GAD2::IRES-Cre (Jackson Laboratory, stock number: *010802*, strain code: Gad2^tm2(cre)Zjh^/J), VGLUT3-Cre (Jackson Laboratory, stock number: 018147, strain code: Tg(Slc17a8-icre)1Edw/SealJ), TH-Cre (Jackson Laboratory, stock number: 008601, strain code: B6.Cg-Tg(Th-cre)1Tmd/J), SERT-Cre (Mouse Mutant Resource and Research Centers, stock number: 017260-UCD, strain code: Tg(Slc6a4-cre)ET33Gsat/Mmucd), ePET-Cre (Jackson Laboratory, stock number: 012712, strain code: B6.Cg-Tg(Fev-cre)1Esd/J) and PITX3-Cre^43^. All lines have been crossed onto the C57BL/6 background for at least six generations. Mice were maintained on a 12:12 light cycle (lights on at 07:00). All procedures complied with the animal care standards set forth by the National Institutes of Health and were approved by University of California, Berkeley’s Administrative Panel on Laboratory Animal Care.

### Stereotaxic injections

Injections were performed under general ketamine–dexmedetomidine anesthesia using a stereotaxic instrument (Kopf Instruments, Model 1900). For red fluorescent retrobead labeling, mice were injected unilaterally with fluorescent retrobeads (LumaFluor Inc.) into the central nucleus of the amygdala (CeA; bregma: −1.22 mm, lateral: 2.8 mm, ventral: −4.7 mm) using a 1 µl Hamilton syringe (Hamilton). The adeno-associated viruses (AAVs) used in this study were from the Deisseroth laboratory (AAV5-EF1α–DIO-hChR2(H134R)-eYFP, AAV5-EF1α-DIO-eYFP; ~10^12^ infectious units per ml, prepared by the University of North Carolina Vector Core) or from the Uchida laboratory (AAV5-flex-RG; AAV5-flex-TVA-mCherry; ~10^12^ infectious units per ml, prepared by the University of North Carolina Vector Core). RV-EnvA-ΔG-GFP was prepared by Kevin T. Beier. For viral injections, concentrated virus solution was injected unilaterally into the DR (bregma: −4.36 mm, lateral: 0 mm, ventral: −3.2 mm), MnR (bregma: −4.36 mm, lateral: 0 mm, ventral: −4.4 mm), VTA (bregma: −3.4 mm, lateral: 0.3 mm, ventral: −4.4 mm) or DR/PAG (two injections with equal volume: bregma: −4.36 mm, lateral: 0 mm, ventral: −3.2 mm and bregma: −4.16 mm, lateral: 0 mm, ventral: −3.2 mm) using a syringe pump (Harvard Apparatus) at 150 nl/min. The injection needle was withdrawn 5 min after the end of the infusion, and the animal was kept on a heating pad until it recovered from anesthesia. Experiments were performed 4–6 weeks (for AAVs) or 7 days (for retrobeads or rabies virus) after stereotactic injection. Injection sites were confirmed in all animals by preparing coronal sections (50 or 100 µm). Injection volumes for each experiment are specified in the main text.

### Histology, immunofluorescence, and confocal microscopy

Mice were transcardially perfused with 4% PFA in PBS, pH 7.4, and brains were post-fixed overnight, and coronal brain slices (50, 75, or 100 μm) were subsequently prepared. For immunohistochemistry, the primary antibodies used were rabbit anti-tyrosine hydroxylase (TH; 1:1000, Millipore), mouse anti-TH (1:1000, Millipore), rabbit anti-tryptophan hydroxylase 2 (TpH; 1:1000, Millipore), goat anti-serotonin (1:1000, Immunostar), and chicken anti-GFP (1:1000, Abcam). The secondary antibodies used were Alexa Fluor 546 goat anti-rabbit, Alexa Fluor 647 goat anti-mouse, Alexa Fluor 488 goat anti-chicken and Alexa Fluor 488 donkey anti-goat (all 1:750, Molecular Probes). Image acquisition was performed with Zeiss LSM510, LSM710, and Nikon A1 laser scanning confocal microscopes, and on a Zeiss AxioImager M2 upright widefield fluorescence/differential interference contrast microscope with charge-coupled device camera using a ×5 objective. Confocal images were analyzed using ImageJ and the Zeiss LSM Image Browser software.

For anatomical characterization and quantification of eYFP-expressing neurons in the VTA, DR and MnR using different transgenic mouse lines, 50 µm coronal sections of the brain area of interest were collected (VTA, bregma: −2.92 to −3.88 mm; DR, bregma: −4.24 to −4.96 mm; MnR, bregma: −4.04 to −4.96 mm) and every other section was analyzed. In the case of the DR, this corresponds to a maximum of ~8 possible sections that could be analyzed per mouse spanning the entire rostrocaudal extent of the DR. Because we sought to characterize Cre expression along the entire rostrocaudal extent of the DR while allowing for small uncontrollable differences in viral spread between animals, animals exhibiting eYFP expression in fewer than 6/8 sections analyzed were excluded from further analysis (*n* = 1 DAT-Cre mouse was excluded accordingly). In every section analyzed, the DR was divided into four subregions measuring ~300 × 300 µm and one confocal image covering ~230 × 230 µm was acquired in each subregion that contained at least one eYFP+ cell. All sections were labelled relative to bregma according to landmarks and nomenclature as described in “*The Mouse Brain in Stereotaxic Coordinates*”^53^. The total number of eYFP-expressing TpH−immunopositive and TpH−immunonegative, or eYFP-expressing TH-immunopositive, and TH-immunonegative, neurons was calculated for each of the four subregions. The same methodological approach was used for quantification of retrogradely labeled (i.e., bead-containing) neurons in the DR/PAG complex (bregma: −4.04 to 4.96 mm; Fig. 6a). To compare cell-type specificity between the anterior and posterior DR, the same analysis was performed on the two (consecutive) most rostral and the two (consecutive) most caudal DR sections for a section of SERT-Cre and ePET-Cre mice.

The anatomical characterization and quantification of eYFP-expressing neurons in the VTA using PITX3-Cre mice (Supplementary Fig. 1s–z) followed an analogous procedure. 50 µm thick coronal sections of the midbrain were subdivided into subregions measuring ~300 × 300 µm and one confocal image covering ~230 × 230 µm was acquired in each midbrain subregion that contained at least one eYFP+ cell. In the anterior midbrain (−3.3 to −2.9 mm from bregma), three subregions were analyzed: one in the lateral VTA which contained the parabrachial pigmented nucleus (PBP), and one at each of two dorsoventral levels in the medial VTA that included the IF and RLi nuclei. In the posterior midbrain, (−3.9 to −3.4 mm from bregma) the lateral subregion again corresponded to the PBP, but medial subregions now encompassed the IF and CLi nuclei, and IPN was included as a fourth subregion comprising the rostral, caudal, intermediate, lateral, dorsolateral, and dorsomedial interpeduncular subnuclei. All sections were labelled relative to bregma according to landmarks and nomenclature as described in “*The Mouse Brain in Stereotaxic Coordinates*”^53^. The total number of eYFP-expressing TH-immunopositive and TH-immunonegative neurons was calculated for each subregion.

### Transsynaptic rabies virus tracing

We used a rabies virus-based genetic mapping strategy^68^ to label presynaptic inputs onto designated starter cell populations, and quantified input cell data using a customized, semi-automated whole-brain mapping MATLAB script^52^. Specifically, DAT-Cre, PITX3-Cre, SERT-Cre and ePET-Cre mice were injected with AAV-FLEX-TVA-mCherry (i.e., a cellular receptor for subgroup A avian leukosis viruses) and AAV-FLEX-RG (i.e., rabies virus glycoprotein; 800 nl, 1:1) into the DR and three weeks later, 300 nl RV-EnvA-ΔG-GFP (i.e., pseudotyped, glycoprotein-deficient, GFP-expressing rabies virus) was injected into the same region (see “Stereotaxic Injections” for coordinates). Seven days after injection, mice were perfused with 4% paraformaldehyde (PFA) in PBS. For input mapping, 75 µm sections of the whole brain were prepared and scanned using a Zeiss Axio Scan Z1 microscope. Individual slices were aligned using customized Matlab scripts. GFP-positive pixels were identified on the basis of a pixel-intensity threshold in the green channel. False-positive pixels (artifacts) were manually identified and removed. Positive pixels were assigned to different brain areas based on “*The Mouse Brain in Stereotaxic Coordinates*”^53^ (Fig. 4a). The “anterior cortex” included orbitofrontal, prelimbic, and infralimbic cortices; “olfactory nuclei” included the accessory olfactory bulb and the anterior olfactory nucleus; and, “other cortex” and “other thal” included all cortical or thalamic areas, respectively, that were not otherwise described. The olfactory bulb and cerebellum were not analyzed, and the injection site (DR) along with nearby regions where true starter cells (TVA-positive, RG-positive, RV-positive) could not be distinguished from pseudostarter cells (TVA-positive, RG-negative, RV-positive) were excluded from analysis (VTA, CLi, RLi). Pixels per brain area were then represented as a percentage of total input pixels. Twelve brain regions were randomly selected to validate this semi-automated quantification method and a human observer counted GFP-positive cells in these regions. These results demonstrated a high correlation between manual scoring of input neurons by an independent observer and our automated segmentation procedure (Fig. 4b; R^2^ = 0.9434, *n* = 12 brain regions).

### Definitions of anatomical abbreviations

Olf Nuclei: anterior and accessory nuclei of the olfactory bulb, Claustrum: claustrum, Anterior Ctx: prelimbic, infralimbic, and orbitofrontal cortices, Motor Ctx: Motor Cortex, Piriform Ctx: piriform cortex, S.S. Ctx: Somatosensory cortex, Visual Ctx: visual cortex, Other Ctx: all other cortical areas not otherwise defined, NAcCore: nucleus accumbens core, NAcMed: nucleus accumbens medial shell, NAcLat: nucleus accumbens lateral shell, DStr: dorsal striatum, VP: ventral pallidum, GPe: globus pallidus external segment, GPi: globus pallidus internal segment, BNST: bed nucleus of the stria terminalis, SI: substantia innominate, CeM: central amygdaloid nucleus medial division, CeC: central amygdaloid nucleus capsular part, CeL: central amygdaloid nucleus lateral division, BLA: basolateral amygdala, DB: diagonal band, Septum: septum, PVA: paraventricular thalamic nucleus, MHb: medial habenula, LHb: lateral habenula, Other Thal: other thalamic areas not otherwise specified, PVH: paraventricular hypothalamic nucleus, LH: lateral hypothalamus, VMH: ventromedial hypothalamus, STh: subthalamic nucleus, MMB: mammillary body, PO: preoptic area, ZI: zona incerta, SN: substantia nigra, DpMe: deep mesencephalic nucleus, Sup. Col.: superior colliculus, PAG: periaqueductal gray, IPN: interpeduncular nucleus, MnR: median raphe, PPTg: pedunculopontine tegmental nucleus, LDTg: laterodorsal tegmental nucleus, PBN: parabrachial nucleus, PnR: pontine reticular nucleus, RMg: raphe magnus nucleus, Amy: amygdala, Ext Amy: extended amygdala, Hypothal: hypothalamus.

### Axon projection analysis

Fifty micrometer sections were immunostained for eYFP and imaged on an Olympus VS120 slide scanning microscope using identical microscope settings across all samples. Images were background subtracted and then binarized based on a pixel-intensity threshold that was held constant for all samples analyzed. Regions of interest were then drawn manually and were defined based on DAPI signal with reference to “*The Mouse Brain in Stereotaxic Coordinates*”^53^. Axon density is reported as ((black pixels in region of interest) / (total pixels in region of interest))*100.

### Electrophysiology

Mice were deeply anaesthetized with pentobarbital (200 mg/kg ip; Vortech). Coronal midbrain slices (200 μm) were prepared after intracardial perfusion with ice-cold artificial cerebrospinal fluid (ACSF) containing (in mM) 50 sucrose, 125 NaCl, 25 NaHCO_3_, 2.5 KCl, 1.25 NaH_2_PO_4_, 0.1 CaCl_2_, 4.9 MgCl_2_, and 2.5 glucose (oxygenated with 95% O_2_/5% CO_2_). After 90 min of recovery, slices were transferred to a recording chamber and perfused continuously at 2–4 ml/min with oxygenated ACSF, containing (in mM) 125 NaCl, 25 NaHCO_3_, 2.5 KCl, 1.25 NaH_2_PO_4_, 11 glucose, 1.3 MgCl_2_ and 2.5 CaCl_2_ at ~30 °C. For recording of excitatory postsynaptic currents (EPSCs), picrotoxin (50 µM, Sigma) was added to block inhibitory currents mediated by GABA_A_ receptors. Cells were visualized with a 40x water-immersion objective on an upright fluorescent microscope (BX51WI; Olympus) equipped with infrared-differential interference contrast video microscopy and epifluorescence (Olympus). Patch pipettes (3.8-4.4 MΩ) were pulled from borosilicate glass (G150TF-4; Warner Instruments) and filled with internal solution, which consisted of (in mM) 117 CsCH_3_SO_3_, 20 HEPES, 0.4 EGTA, 2.8 NaCl, 5 TEA, 4 MgATP, 0.3 NaGTP, 5 QX314 and 0.1% neurobiotin, pH 7.35 (270–285 mOsm). Electrophysiological recordings were made using a MultiClamp700B amplifier and acquired using a Digidata 1550 digitizer, sampled at 10 kHz, and filtered at 2 kHz. All data acquisition was performed using pCLAMP software (Molecular Devices). Channelrhodopsin-2 (ChR2) was stimulated by flashing 473 nm light through the light path of the microscope using an ultrahigh-powered light-emitting diode (LED) powered by an LED driver (Prizmatix) under computer control. A dual lamp house adaptor (Olympus) was used to switch between fluorescence lamp and LED light source. The light intensity of the LED was not changed during the experiments and the whole slice was illuminated (5 mW/mm^2^). Light-evoked excitatory postsynaptic currents (EPSCs) were obtained every 10 s with one pulse of 473 nm light (5 ms) with neurons voltage clamped at −70 mV. Series resistance (15–25 MΩ) and input resistance were monitored online. For pharmacological experiments, we recorded baseline responses for 3 min and bath applied 10 μM CNQX (Tocris) for 5–10 min to block AMPA/kainate receptor mediated currents. Data were analyzed offline using IgorPro Software (Wavemetrics). Light-evoked EPSC amplitudes were calculated by averaging responses from 10 sweeps and then measuring the peak amplitude in a 50 ms window after the light pulse. Cells that did not show a peak in this window that exceeded the baseline noise (10 pA) were classified as non-responders.

### Statistics

Data from electrophysiology, mouse line characterization, and anatomical tracing experiments that met assumptions of equal variance were analyzed using two-tailed *t*-tests, one-way, and two-way ANOVAs using GraphPad Prism 6 (Graphpad Software). For data that did not meet the equal variance assumption (Spearman’s test) we applied a Box-Cox transformation with lambda values determined using RStudio, and then performed statistical analysis on transformed values as described above. For data that failed to meet the equal variance assumption even after a Box-Cox transformation, we compared group means directly using heteroscedastic *t*-tests. Wherever multiple comparisons were made, the false discovery rate was held at the 0.05 level using the Benjamini-Hochberg procedure and reported *p*-values are FDR-adjusted. Statistical significance was *p < 0.05, **p < 0.01, ***p < 0.001. All data are presented as means ± SEM.

### Reporting summary

Further information on research design is available in the Nature Research Reporting Summary linked to this article.

## Supplementary information

Supplementary Information

Reporting Summary

Source Data

## Data Availability

The source data underlying Figs. 1c–d, f–g, i–j, l–m, o–t, 2c-e, h, j, 3b-c, 4b, e, 5, 6b, d–f; and Supplementary Fig. 1f-g, j-k, o-n, r, t, v, x, y-z are provided as a Source Data file.

## Code availability

Custom code used for analysis in this manuscript is available upon reasonable request.
