# Supplementary material for: Characterization of transgenic mouse models targeting neuromodulatory systems reveals organizational principles of the dorsal raphe
Source: Nat Commun. 2019 Oct 11;10:4633. doi: 10.1038/s41467-019-12392-2 (PMC6789139; doi:10.1038/s41467-019-12392-2)

SUPPLEMENTARY FIGURE LEGENDS

Supplementary Figure 1. Control experiments and additional data for characterization of transgenic mouse lines targeting DA and 5HT neurons

(a) Schematic of experimental design showing viral injection into the DR.

(b) Confocal image showing minimal eYFP expression when AAV-DIO-eYFP virus was injected into a wildtype mouse (scale bar 0.5 mm).

(c) Confocal image showing numerous eYFP+ cells when the same virus in (b) was injected into a GAD2-Cre mouse (scale bar 0.5 mm).

(d) Confocal overview image showing colocalization between immunohistochemically detected TpH and immunohistochemically detected 5HT (scale bar 0.5 mm).

(e) High magnification confocal image showing near perfect colocalization between TpH and 5HT immunolabeling in the DR (scale bar 50 μm).

(f-g) Vertical slice charts showing percentage of 5HT+ neurons that are TpH+ (f) and vice versa (g).

(h) Schematic of experimental design showing viral injection into the median raphe (MnR) of SERT-Cre mice.

(i) Confocal image showing colocalization of eYFP (green) and tryptophan-hydroxylase 2 (TpH) immunopositive (red) neurons in the MR, which is subdivided into three different subregions (scale bar 1 mm).

(j) Sample confocal images showing eYFP and TpH-positive neurons for the different areas delineated in (b). Vertical slice charts indicate percentage of eYFP-positive cells that co-express TpH (eYFP+ TpH+, blue) or lack expression of TpH (eYFP+ TpH-, orange) for individual MR subregions (scale bar 50 μm).

(k) Pie chart showing total percentage of eYFP+ and TpH+ (blue) and eYFP+ and TpH- (orange) cells when the three MnR subregions are considered collectively.

(l-o) Same as (h-k) but for anatomical characterization of ePET-Cre mice in the MnR.

(p) Schematic of experimental design showing large (1 μl) viral injection into the DR of PITX3-Cre mice.

(q) Confocal image showing colocalization between eYFP (green) and TH- immunopositive (red) cells in the DR. Note prominent eYFP expression outside the DR (scale bar 1 mm).

(r) Pie chart showing total percentage of eYFP+ and TH+ (blue) and eYFP+ and TH- (orange) cells in the DR of PITX3-Cre mice.
(s) Schematic of experimental design showing viral injection into the ventral tegmental area (VTA) of PITX3-Cre mice.
(t) Pie charts showing the total percentage colocalization of eYFP with TH-immunopositive (blue) or TH-immunonegative (orange) cells for the entire ventral midbrain of PITX3-Cre mice.
(u, w) Representative fluorescence images of coronal brain slices showing TH-immunostaining (red) and eYFP-expression (green) in the rostral (u) and caudal (w) midbrain from a PITX3-Cre mouse (scale bar 1 mm).
(v, x) Pie charts showing the total percentage colocalization of eYFP with TH-immunopositive (blue) or TH-immunonegative (orange) cells in the rostral (v) or caudal (x) midbrain of PITX3-Cre mice.
(y, z) Confocal images showing TH-immunostaining (red) and eYFP-expression (green) for the different areas delineated in (u) and (w). Note the low cell-type specificity observed in midline VTA subregions which include the interfascicular and rostral linear nuclei. Vertical slice charts indicate percentage of eYFP-positive cells that co-express TH (eYFP+ TH+, blue) or lack expression of TH (eYFP+ TH-, orange) for individual VTA subregions (scale bar 50 µm). Data provided as a Source Data file.

Supplementary Figure 2. Topography of genetically identified DR cell populations for individual mice.
Sample confocal images of the DR from mice used in the analyses shown in Figure 1 and Figure 2. Note the consistent anatomical distribution patterns of genetically identified DR cell populations across mice from the same mouse line. Asterisks indicate images that have been reproduced from Figures 1 and 2 which are shown again here to facilitate a comparison of the anatomical distribution of eYFP labeled cells between mice of the same genotype (eYFP: green, TH-immunostaining: blue, TpH-immunostaining: red; scale bars 0.2 mm).

Supplementary Figure 3. Projections of genetically identified DR cell populations.
(a) Background subtracted and thresholded images showing axon fibers from eYFP-labeled DR neurons innervating the VTA and amygdala. Note that projections from DR 5HT, DA, glutamate

and GABA neurons innervate the VTA relatively evenly, but favor specific subnuclei of the amygdala (scale bar 0.2 mm).

(b) Schematic showing the preferential (but not exclusive) projection targets in the amygdala of distinct DR cell populations (vlPAG: ventrolateral periaqueductal gray, dDR: dorsal dorsal raphe, vlDR: ventrolateral dorsal raphe, vDR: ventral dorsal raphe, BLA: basolateral amygdala, CeL: central amygdaloid nucleus lateral division, CeM: central amygdaloid nucleus medial division, CeC: central amygdaloid nucleus capsular part).

Supplementary Figure 4. Control experiments for mapping monosynaptic inputs onto DR neurons.

(a) Schematic of viral injections into wildtype (C57Bl/6) mice.

(b) Sample fluorescence image of a coronal brain slice showing very few GFP-positive cells in the DR. Note that subregion #1 contains two GFP-positive cells (scale bar 0.5 mm).

(c) Schematic of viral injections into SERT-Cre mice. Note that helper AAVs were omitted in this control.

(d) Sample fluorescence image of a coronal brain slice showing very few GFP-positive cells in the DR. Note that subregion #3 contains two GFP-positive cells (scale bar 0.5 mm).

(e) Schematic of viral injections into SERT-Cre mice. Note that AAV-FLEX-RG was omitted in this control.

(f) Sample fluorescence image of a coronal brain slice showing GFP+ (i.e. RV+) cells in the DR (scale bar 0.5 mm).

(g-i) Sample fluorescence images showing brain regions known to send inputs to DR 5HT neurons. Note the lack of GFP labeled cells in these images, confirming that there is no transsynaptic spread of the RV in the absence of RG (scale bar 1 mm).

Supplementary Fig. 1 Cardozo Pinto et al.

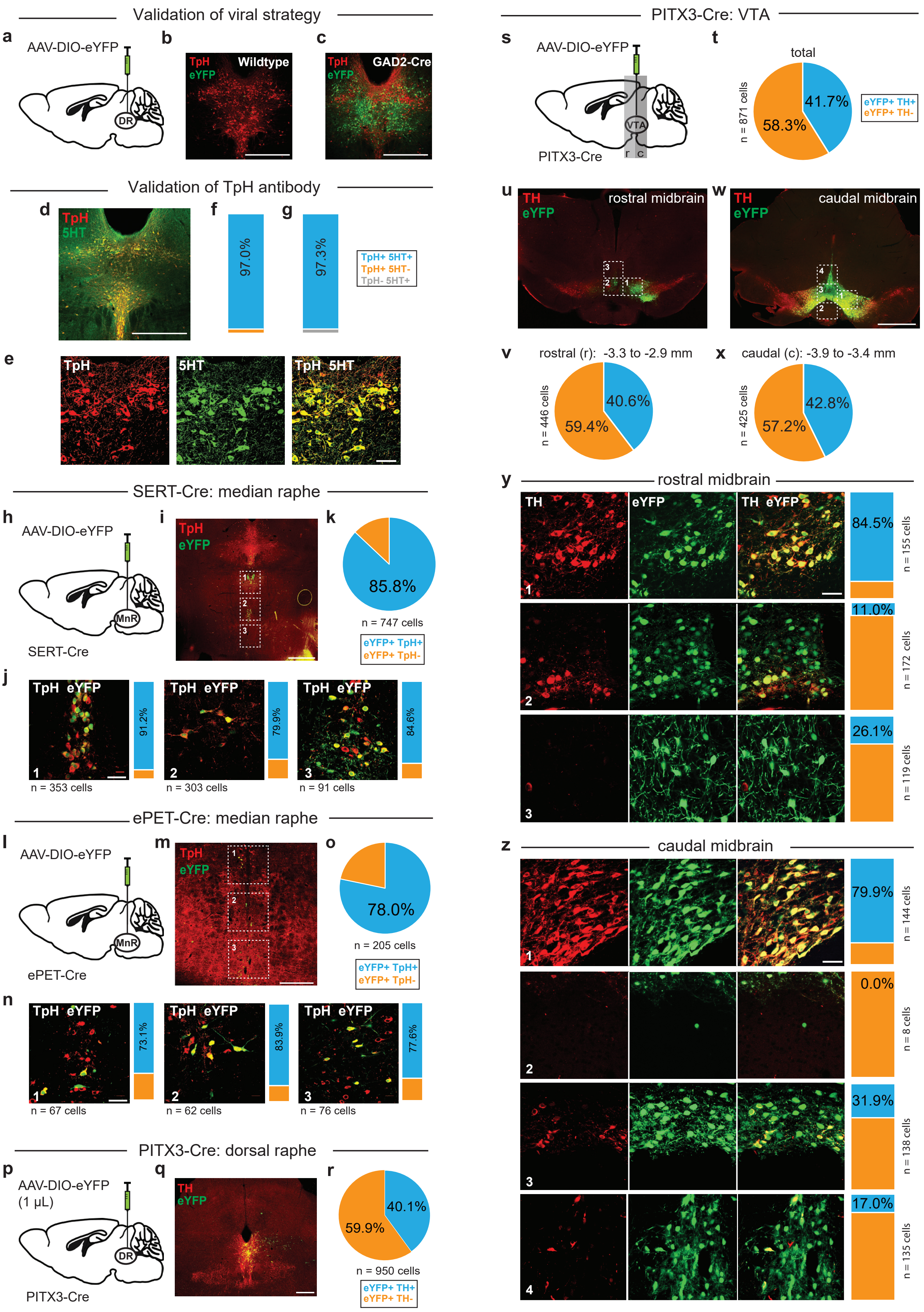

Supplementary Fig. 2 Cardozo Pinto et al.

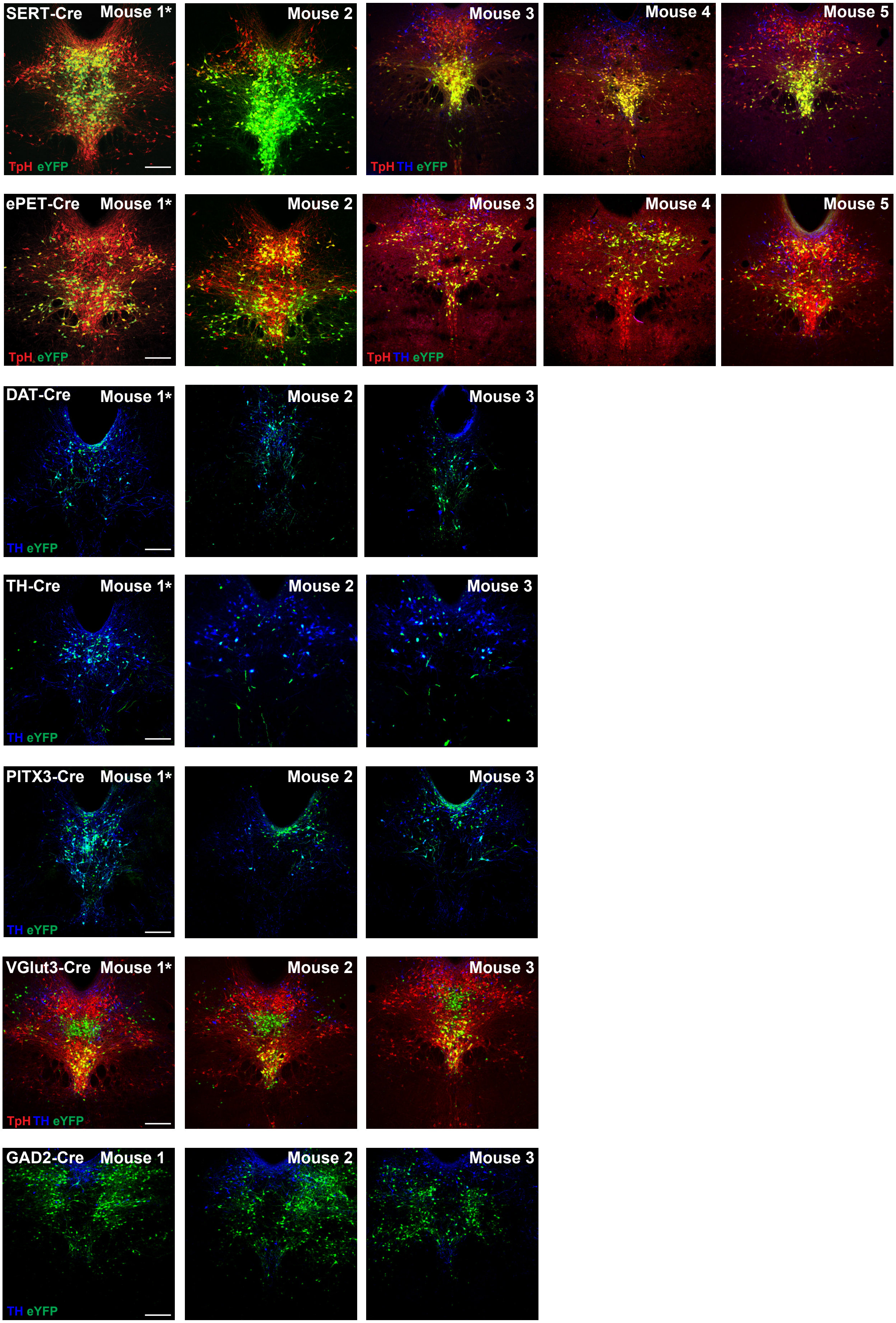

Supplementary Fig. 3 Cardozo Pinto et al.

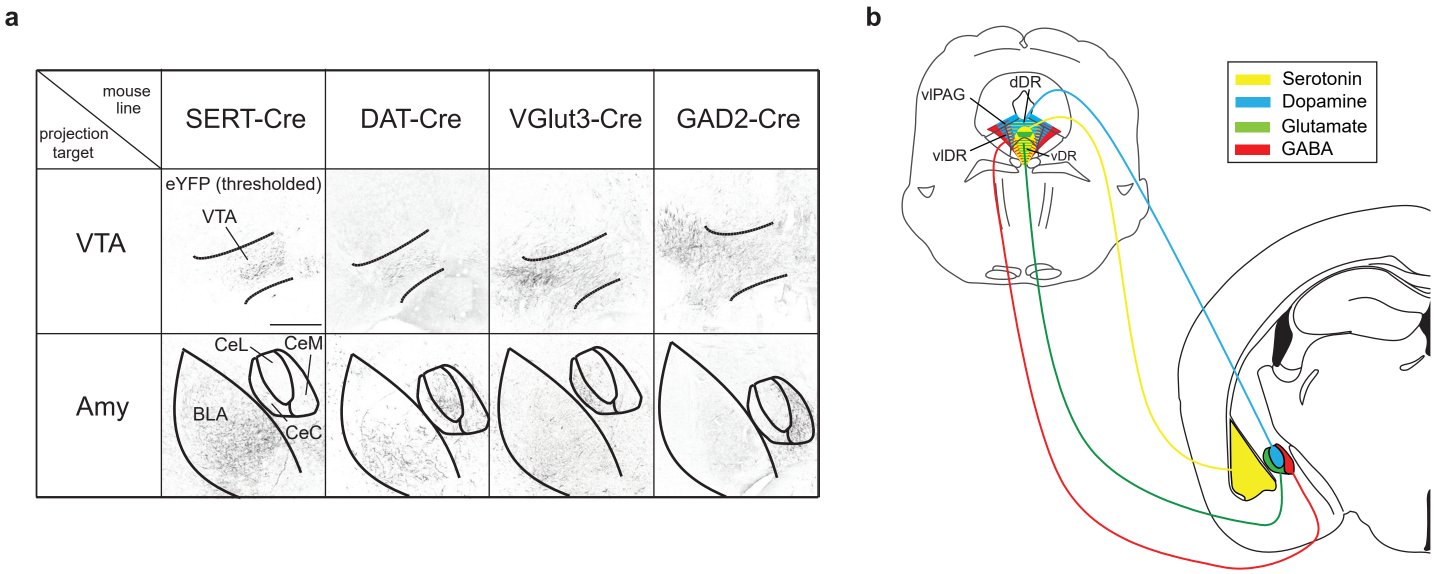

Supplementary Fig. 4 Cardozo Pinto et al.

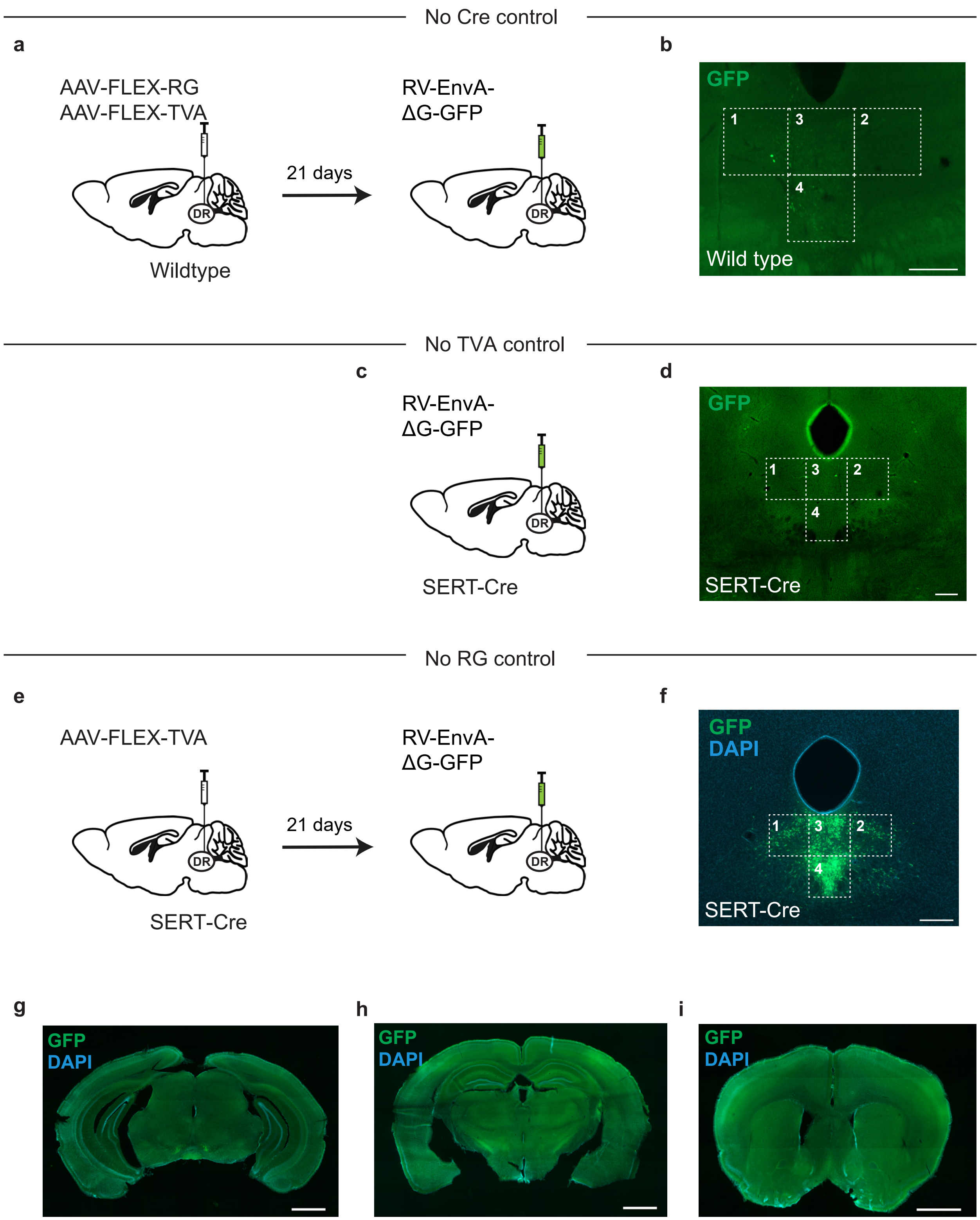

Supplement: Supplementary file 1 — Supplementary Information [file 41467_2019_12392_MOESM1_ESM.pdf]
