# Supplementary material for: Characterization of transgenic mouse models targeting neuromodulatory systems reveals organizational principles of the dorsal raphe
Source: Nat Commun. 2019 Oct 11;10:4633. doi: 10.1038/s41467-019-12392-2 (PMC6789139; doi:10.1038/s41467-019-12392-2)
Supplement: Supplementary file 2 — Reporting Summary [file 41467_2019_12392_MOESM2_ESM.pdf]

nature research

Corresponding author(s): Stephan Lammel

Last updated by author(s): Jul 5, 2019

Reporting Summary

Nature Research wishes to improve the reproducibility of the work that we publish. This form provides structure for consistency and transparency in reporting. For further information on Nature Research policies, see Authors & Referees and the Editorial Policy Checklist.

Statistics

For all statistical analyses, confirm that the following items are present in the figure legend, table legend, main text, or Methods section.

n/a	Confirmed	
☐	☒	The exact sample size (*n*) for each experimental group/condition, given as a discrete number and unit of measurement
☐	☒	A statement on whether measurements were taken from distinct samples or whether the same sample was measured repeatedly
☐	☒	The statistical test(s) used AND whether they are one- or two-sided<br>*Only common tests should be described solely by name; describe more complex techniques in the Methods section.*
☒	☐	A description of all covariates tested
☐	☒	A description of any assumptions or corrections, such as tests of normality and adjustment for multiple comparisons
☐	☒	A full description of the statistical parameters including central tendency (e.g. means) or other basic estimates (e.g. regression coefficient) AND variation (e.g. standard deviation) or associated estimates of uncertainty (e.g. confidence intervals)
☐	☒	For null hypothesis testing, the test statistic (e.g. *F*, *t*, *r*) with confidence intervals, effect sizes, degrees of freedom and *P* value noted<br>*Give P values as exact values whenever suitable.*
☒	☐	For Bayesian analysis, information on the choice of priors and Markov chain Monte Carlo settings
☒	☐	For hierarchical and complex designs, identification of the appropriate level for tests and full reporting of outcomes
☒	☐	Estimates of effect sizes (e.g. Cohen's *d*, Pearson's *r*), indicating how they were calculated

Our web collection on statistics for biologists contains articles on many of the points above.

Software and code

Policy information about availability of computer code

Data collection	PClamp 10 (Molecular Devices) was used for acquisition of electrophysiology data. Zen Software (Zeiss) were used for acquiring fluorescence and confocal images.
Data analysis	GraphPad Prism 6 (Graphpad Software) was used for statistical analysis. ImageJ (NIH) and Zeiss LSM Image Browser were used for analysis of fluorescence and confocal images. IgorPro Software was used for analysis of electrophysiology data (Wavemetrics). Custom matlab software (previously published, de Jong et al. 2019, Neuron) was used for analysis of rabies tracing data.

For manuscripts utilizing custom algorithms or software that are central to the research but not yet described in published literature, software must be made available to editors/reviewers. We strongly encourage code deposition in a community repository (e.g. GitHub). See the Nature Research guidelines for submitting code & software for further information.

Data

Policy information about availability of data

All manuscripts must include a data availability statement. This statement should provide the following information, where applicable:

- Accession codes, unique identifiers, or web links for publicly available datasets
- A list of figures that have associated raw data
- A description of any restrictions on data availability

The data that support the findings of this study are available from the corresponding author upon reasonable request.

Field-specific reporting

Please select the one below that is the best fit for your research. If you are not sure, read the appropriate sections before making your selection.

☒ Life sciences ☐ Behavioural & social sciences ☐ Ecological, evolutionary & environmental sciences

For a reference copy of the document with all sections, see nature.com/documents/nr-reporting-summary-flat.pdf

Life sciences study design

All studies must disclose on these points even when the disclosure is negative.

Sample size	No statistical methods were used to predetermine sample size, which were based on work in previous publications (Lammel et al., 2015).
Data exclusions	To ensure that our mouse line characterization experiments sampled the entire rostrocaudal extent of the DR, we sectioned the brain at 50 um, collected every other slice, and excluded any animals in which fewer than six (out of a possible total of 8) DR sections contained eYFP+ cells. n=1 DAT-Cre mouse was excluded and replaced for not meeting this criterion.
Replication	Experiments were repeated so that our data are based on at least on two independent experiments with similar results with exception of data shown in Supplementary Fig. 1A-C and 1D-G, which was performed in n = 1 mouse.
Randomization	For all experiments in this study mice were assigned to groups based on genotype and consequently randomization was not used.
Blinding	Blinding was not used in the experiments in this study.

Reporting for specific materials, systems and methods

We require information from authors about some types of materials, experimental systems and methods used in many studies. Here, indicate whether each material, system or method listed is relevant to your study. If you are not sure if a list item applies to your research, read the appropriate section before selecting a response.

Materials & experimental systems

n/a	Involved in the study	
☐	☒	Antibodies
☒	☐	Eukaryotic cell lines
☒	☐	Palaeontology
☐	☒	Animals and other organisms
☒	☐	Human research participants
☒	☐	Clinical data

Methods

n/a	Involved in the study	
☒	☐	ChIP-seq
☒	☐	Flow cytometry
☒	☐	MRI-based neuroimaging

Antibodies

Antibodies used	The primary antibodies used were rabbit anti-tyrosine hydroxylase (TH; 1:1000, Millipore), mouse anti-TH (1:1000, Millipore), rabbit anti-tryptophan hydroxylase 2 (TpH; 1:1000, Millipore), goat anti-serotonin (1:1000, Immunostar) and chicken anti-GFP (1:1000, Abcam). The secondary antibodies used were Alexa Fluor 546 goat anti-rabbit, Alexa Fluor 647 goat anti-mouse, Alexa Fluor 488 goat anti-chicken and Alexa Fluor 488 donkey anti-goat (all 1:750, Molecular Probes).
Validation	TH antibodies have been extensively validated in previous studies (refs. Lammel et al., 2008; Lammel et al., 2015). The TpH2 antibody was validated by analyzing the overlap between 5HT and TpH2 immunostaining in the dorsal raphe (Supplementary Fig. S1D-G). The GFP antibody has been used in over 1000 published studies (see webpage for Abcam #ab13970).

Animals and other organisms

Policy information about studies involving animals; ARRIVE guidelines recommended for reporting animal research

Laboratory animals	The following mouse lines (25-30 g, 8-14 weeks old, male) were used for experiments: C57BL/6J mice (Jackson Laboratory, stock number: 000664), DAT::IRES-Cre (Jackson Laboratory, stock number: 006660, strain code: B6.SJL-Slc6a3tm1.1(cre)Bkmn/J), VGLUT2::IRES-Cre (Jackson Laboratory, stock number: 016963, strain code: Slc17a6tm2(cre)Lowl/J), GAD2::IRES-Cre (Jackson Laboratory, stock number: 010802, strain code: Gad2tm2(cre)Zjh/J), VGLUT3-Cre (Jackson Laboratory, stock number: 018147, strain code: Tg(Slc17a8-icre)1Edw/SealJ), TH-Cre (Jackson Laboratory, stock number: 008601, strain code: B6.Cg-Tg(Th-cre)1Tmd/J), SERT-Cre (Mouse Mutant Resource and Research Centers, stock number: 017260-UCD, strain code: Tg(Slc6a4-cre)ET33Gsat/Mmucd), ePET-Cre (Jackson Laboratory, stock number: 012712, strain code: B6.Cg-Tg(Fev-cre)1Esd/J) and PITX3-Cre.

Wild animals	Not applicable.
Field-collected samples	Not applicable.
Ethics oversight	All procedures complied with the animal care standards set forth by the National Institutes of Health and were approved by University of California, Berkeley's Administrative Panel on Laboratory Animal Care.

Note that full information on the approval of the study protocol must also be provided in the manuscript.
